# *Roodmus*: a toolkit for benchmarking heterogeneous electron cryo-microscopy reconstructions

**DOI:** 10.1107/S2052252524009321

**Published:** 2024-10-15

**Authors:** Maarten Joosten, Joel Greer, James Parkhurst, Tom Burnley, Arjen J. Jakobi

**Affiliations:** a Department of Bionanoscience, Kavli Institute of Nanoscience Delft University of Technology 2629 HZDelft The Netherlands; b Science and Technology Facilities Council Research Complex at Harwell OxonOX11 0FA United Kingdom; c Rosalind Franklin Institute, Harwell Science and Innovation Campus, OxonOX11 0QS, United Kingdom; d Diamond Light Source Harwell Science and Innovation Campus OxonOX11 0DE United Kingdom; Max Planck Institute of Molecular Physiology, Germany

**Keywords:** cryo-EM, biological macromolecules, *Roodmus*, single-particle averaging, heterogeneous reconstruction algorithms, molecular dynamics simulations, SARS-CoV-2, conformational heterogeneity, conformational trajectories

## Abstract

Conformational heterogeneity of biological macromolecules is a challenge in single-particle averaging (SPA). Current standard practice is to employ classification and filtering methods that may allow a discrete number of conformational states to be reconstructed. However, the conformation space accessible to these molecules is continuous and, therefore, explored incompletely by a small number of discrete classes. Recently developed heterogeneous reconstruction algorithms (HRAs) to analyse continuous heterogeneity rely on machine-learning methods that employ low-dimensional latent space representations. The non-linear nature of many of these methods poses a challenge to their validation and interpretation and to identifying functionally relevant conformational trajectories. These methods would benefit from in-depth benchmarking using high-quality synthetic data and concomitant ground truth information. We present a framework for the simulation and subsequent analysis with respect to the ground truth of cryo-EM micrographs containing particles whose conformational heterogeneity is sourced from molecular dynamics simulations. These synthetic data can be processed as if they were experimental data, allowing aspects of standard SPA workflows as well as heterogeneous reconstruction methods to be compared with known ground truth using available utilities. The simulation and analysis of several such datasets are demonstrated and an initial investigation into HRAs is presented.

## Abstract 2

*Roodmus* is a toolkit sourcing conformational heterogeneity of biomacromolecules from molecular dynamics simulations to generate high-quality synthetic data for the development and benchmarking of heterogeneous reconstruction algorithms.

## 1. Introduction

Cryogenic sample electron microscopy (cryo-EM) has become an established method to determine 3D structures of biological macromolecules at resolutions where atomic interpretation becomes possible (Kühlbrandt, 2014 ▸; Egelman, 2016 ▸). The success of the field has largely been attributed to direct electron detectors, improved stability of electron optical components as well as advanced image processing software (Nogales, 2016 ▸; Cheng, 2015 ▸). One of the remaining challenges in the field is to improve methods for extracting the structural heterogeneity inherent in cryo-EM data to provide meaningful functional insights.

In single-particle averaging (SPA), samples consist of a suspension of biological macromolecules in solution which is then vitrified by flash-freezing in liquid ethane. As a result, we presume each particle has a different orientation and conformation. Although the vitrification of the sample is not instantaneous, leaving time for molecules to anneal into a lower-energy state, recent work suggests that a sizeable portion of the conformational states of the protein at room temperature is still populated in the vitrified state (Bock & Grubmüller, 2022 ▸).

The final result of an SPA workflow is often a single-consensus 3D reconstruction representing the macromolecule of interest. In such a consensus map, the conformational states of the particle are averaged, which causes the density, particularly in flexible regions, to be blurred and effectively reduces local resolution. It is therefore standard practice to employ strategies to reduce the heterogeneity in the data by means of discrete 3D classification into structurally homogeneous particle subsets (Scheres, 2016 ▸). In cases where the heterogeneity is limited to a discrete set of distinctly different conformations, this strategy can be used to obtain structures of these complexes in their different structural states (Nguyen *et al.*, 2016 ▸; Zhou *et al.*, 2020 ▸).

However, subdivision of the cryo-EM data into a discrete number of subsets is not well suited for the description of continuous types of molecular motion, for which an infinite number of subsets would, in principle, be needed. In recent years, methods have been developed that aim to more comprehensively explore the conformational heterogeneity present in cryo-EM data. Various strategies have been used to accomplish this goal, which we collectively call heterogeneous reconstruction algorithms (HRAs). Review articles discussing these approaches are available (Sorzano *et al.*, 2019 ▸; Toader *et al.*, 2023 ▸; Serna, 2019 ▸; Tang *et al.*, 2023 ▸; Sorzano, 2024 ▸), so here we discuss a selection of methods.

One approach is to use principal component analysis (PCA) to determine which transformations maximize the statistical variance in the data. One such technique, multi-body refinement (Nakane *et al.*, 2018 ▸), builds on the assumption that large-scale conformational changes of a complex can be described by a discrete number of independently moving rigid bodies that themselves are identical across the particle images. After alignment of the individual bodies using signal subtraction and focused refinement, PCA on the relative orientations of all bodies for every experimental image is used to characterize the most dominant motions in the complex. This method is limited by requiring the user to define rigid bodies sizeable enough for refinement within the structure of interest. The estimated conformational heterogeneity is also limited to large domain motion.

Normal mode analysis can also be used to provide a linear interpretation of the heterogeneity present. *HEMNMA* (Jin *et al.*, 2014 ▸) is one such example which performs an iterative 3D (reference map) to 2D (projected particle image) elastic and rigid body alignment to find the conformation, orientation and translation quasi-simultaneously. Extensions to this method include the use of neural networks for increasing the speed of processing (Hamitouche & Jonic, 2022 ▸), combination with molecular dynamics (MD) (Vuillemot *et al.*, 2022 ▸) and further combination with PCA (Vuillemot *et al.*, 2023 ▸).

Another approach is 3D variability analysis (Punjani & Fleet, 2021 ▸), which uses a form of PCA to decompose the conformational heterogeneity in a set of particle images into principal modes of motion and a per-particle latent vector. The latent vector is a weighting of all modes of motion present in the particle image. This method offers a way to represent continuous conformational heterogeneity as a continuous manifold, similar to *ManifoldEM* approaches (Dashti *et al.*, 2014 ▸; Frank & Ourmazd, 2016 ▸; Dashti *et al.*, 2020 ▸).

Numerous other approaches also aim to embed all 2D projected particle images into a low-dimensional latent space that encodes the heterogeneity in the data by utilizing variational autoencoders (VAEs). *CryoDRGN* (Zhong *et al.*, 2021 ▸) is an example of such a method, where the encoder and decoder are both implemented as convolutional neural networks and the decoder directly outputs a 3D volume density representation. Although for most of these methods the encoder model is similar, there are different choices for the implementation of the decoder model. *3DFlex* (Punjani & Fleet, 2023 ▸) outputs a deformation field that specifies the way a consensus volume should be deformed to reach a specific state according to the sampled location in the latent space. *DynaMight* and *EMAN2*’s *e2gmm* program opt to model the density as a Gaussian mixture model (Chen & Ludtke, 2021 ▸; Schwab *et al.*, 2023 ▸). The parameters of the mixture model are then the result of passing a latent variable through the decoder.

In many cases these methods are used as a replacement for 3D classification or to filter the particles such that one or more homogeneous datasets remain (Leesch *et al.*, 2023 ▸; Huang *et al.*, 2022 ▸; Serna *et al.*, 2022 ▸; Schoppe *et al.*, 2021 ▸) and the full potential of HRAs to explore conformational heterogeneity in cryo-EM data is not always utilized. It can be challenging and time-consuming to optimize the model and interpret and validate results obtained with deep-learning-based methods. As with any algorithm, the performance of HRAs depends on the hyperparameters used and these may require tuning to achieve the best results. The latent space embedding requires further analysis by clustering or dimensionality-reduction methods to arrive at biologically interpretable results.

We believe HRA development will benefit from thorough benchmarking with known ground truth data. This will allow the assessment of conformation sampling for both completeness of conformation space and relative populations. It will also allow exploration of the effect of hyperparameter optimization and aid in the interpretation of results.

Simulated data have previously been used in demonstrating various heterogeneous reconstruction methods such as *CryoDRGN* and *e2gmm*. In these cases, a simple linear motion of a structure was simulated. Projection images of the structures were then generated, and Gaussian noise was added. Our new approach allows more complex heterogeneity to be modelled by utilizing the conformational states sampled from MD simulations. We use *Parakeet* (Parkhurst *et al.*, 2021 ▸) software to simulate our datasets. *Parakeet* employs a multislice approach to propagate an electron wave through a thin amorphous ice layer in which scattering molecules are placed in random orientations. As these micrographs are suitable for processing using conventional 3D reconstruction workflows, it is possible to gain an increased understanding of how the application of each step in the reconstruction workflow, including particle picking and classification, contributes to the final result. We called this simulation and analysis toolkit *Roodmus*.

In this paper, we first expand on how the *Roodmus* toolkit is designed and how it can be utilized to perform the case studies which are subsequently reported. We then demonstrate the effect of including conformational heterogeneity in a synthetic dataset on a conventional SPA reconstruction workflow. We probe the current limitations of the simulation approach through an investigation into how fluence and a radiation damage model affect the consensus reconstructions. Subsequently, we investigate 3D classification of synthetic data with discrete heterogeneity and explore how conformational heterogeneity is expressed in embeddings of the latent representations provided by two prominent VAE based approaches: *CryoDRGN* and *3DFlex*.

## 2. Results

### 2.1. *Roodmus* workflow

The goal of the *Roodmus* toolkit is to generate realistic datasets of synthetic electron micrographs and allow the ground truth information, including particle positions, particle orientations, particle conformations and electron optical parameters, to be stored and later mapped to particles in the conventional SPA or HRA workflow metadata files.

**Figure 1:**
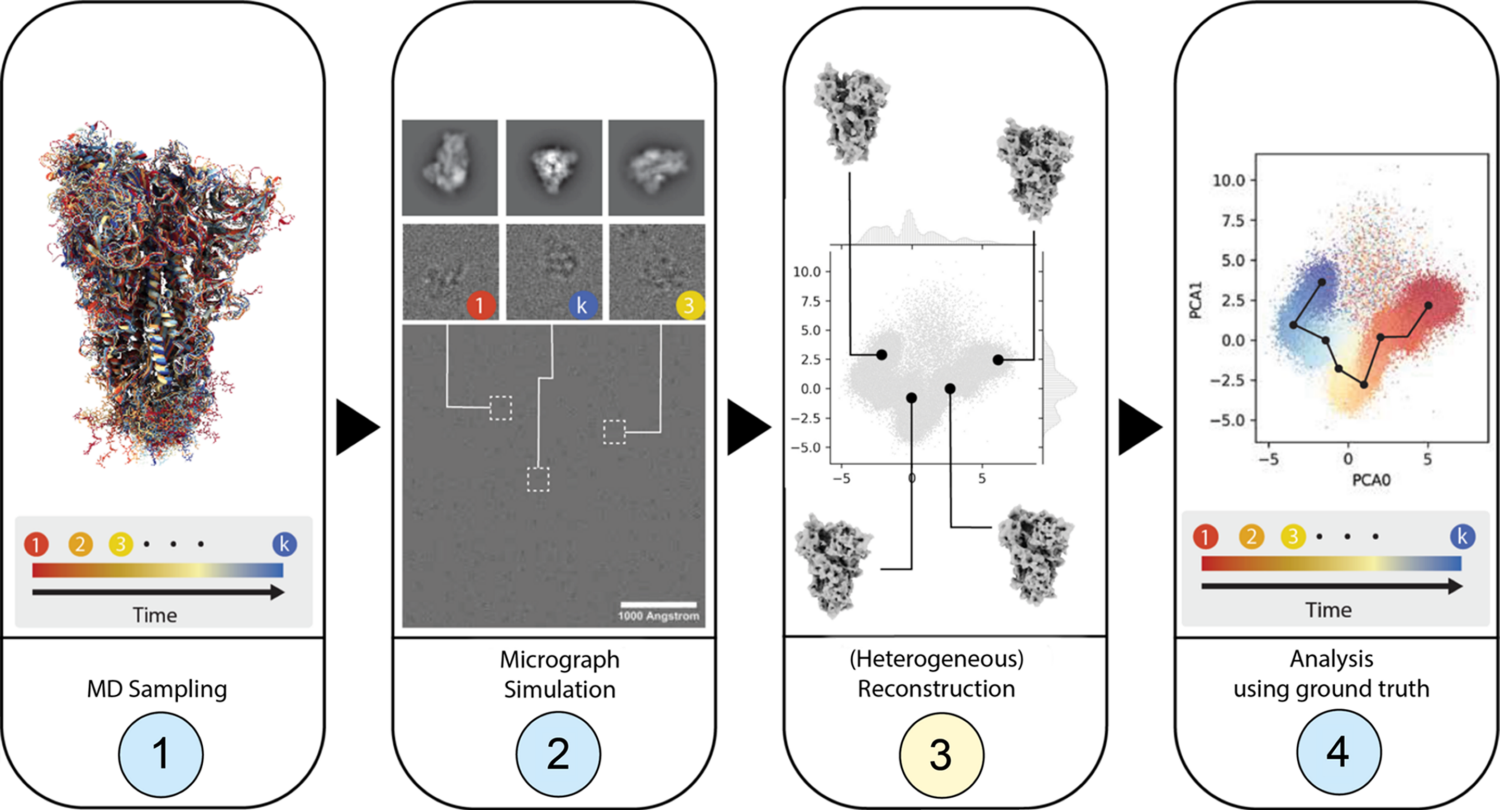
Overview of a four-step workflow that makes use of the *Roodmus* framework. Nodes highlighted in yellow are performed using software external to the *Roodmus* framework; steps in blue use *Roodmus* utilities. (1) A user-provided MD simulation is used as the input to the data generation. (2) Time points from the trajectory may be sampled via *Roodmus*, which subsequently simulates micrographs using the *Parakeet* simulation package in which the conformation of each particle is sampled from these time points. (3) These synthetic micrographs can be processed using 3D reconstruction software, including HRAs. (4) Every step of the resulting reconstruction pipelines can then be analysed using *Roodmus* utilities to compare reconstruction outputs to ground truth information. *Roodmus* currently supports metadata from *CryoSPARC* and *RELION* pipelines as well as *CryoDRGN* and *3DFlex* metadata.

The first step (MD sampling, see Fig. 1 ▸) serves to acquire a source of structural heterogeneity and to sample atomic coordinate models from it. We suggest sourcing a motion trajectory of a biomolecular complex or another structure of interest. Atomistic MD simulations sampled over a sufficiently long time provide the most detailed observable conformational changes, although sampling large domain motion may be too computationally demanding. In these cases, various forms of enhanced sampling can be considered as well, such as steered MD, meta-dynamics or replica-exchange MD (Yang *et al.*, 2019 ▸). In this work, we showcase applications using both atomistic MD trajectories of up to 500 µs as well as steered MD trajectories that force a large conformational change to occur within time frames of 100 and 200 ps. The left-most panel of Fig. 1 ▸ shows an example of a conformational ensemble sampled from a 10 µs MD trajectory of the SARS-CoV-2 spike protein (Shaw, 2020 ▸). Large trajectories that are finely sampled can be downsampled to *k* conformations by selecting equidistant time points using a *Roodmus* utility.

Next, we configure the *Parakeet* simulation package to create synthetic micrographs. A random selection is made out of the available conformations to build a sample. The positions and orientations of each molecule can be specified at this time or they can be randomly generated by *Parakeet*, sampling the orientation of each particle uniformly from the *SO*(3) rotation group. Each particle has a bounding box and cannot overlap with other particles. The sample thickness can also be specified and can cause particles to overlap in projection when the thickness allows for multiple particles to be placed along the projection axis. Other imaging parameters including fluence, acceleration voltage, defocus and electron optical aberrations can be specified. In our investigations, unless otherwise stated, all datasets created were simulated with a total fluence of 45 e^−^ Å^−2^, acceleration voltage of 300 kV and the Gaussian Random Field ice model (Parkhurst *et al.*, 2024 ▸) with an ice thickness of 50 nm. In addition, the defocus used in image simulation is sampled from one or more normal distributions, each with an adjustable mean and variance. This is done to reproduce typical experimental defocus distributions resulting from variations in grid planarity and commonly applied focusing protocols during low-dose imaging. *Parakeet* then propagates the electron wave through the virtual sample and applies the contrast transfer function (CTF) and simulated detector effects to produce a micrograph or dose-fractionated movie.

The synthetic dataset can then be processed using common SPA workflows or HRAs. Ground truth information is saved during simulation, including particle positions, orientations and defocus values. This information can subsequently be compared with the metadata output at any stage of processing. Additionally, this information can be compared with the outputs from HRAs. For example, a latent space representation of heterogeneity can be labelled with the time at which that conformation occurs in the MD simulation, as is shown in panel 4 of Fig. 1 ▸.

In the next sections, we demonstrate the usefulness of applying synthetic data to all aspects of cryo-EM SPA workflows and show the effects of sample heterogeneity on 3D reconstruction. We will conclude with the application of *CryoDRGN* and *3DFlex* to our synthetically generated datasets and compare the results with the known conformational heterogeneity from the simulations.

### 2.2. Effect of increasing heterogeneity in simulated single-particle data

To investigate the impact of heterogeneity on consensus reconstructions, we generated two datasets from a 10 µs MD simulation of the SARS-CoV-2 spike glycoprotein in the partially open conformation (labelled DESRES-ANTON-11021571) (Shaw, 2020 ▸). The trajectory consists of 8334 time points with a 1.2 ns interval. For the first dataset, a single conformation was extracted from this trajectory and used to generate 34 micrographs, containing 10 200 particles in total. The second dataset utilized all conformations from the MD trajectory and consisted of 900 synthetic micrographs containing 270 000 particles in total. We increased the amount of data generated to ensure that the sampled viewing directions for each conformation are sufficient for later analysis.

**Figure 2:**
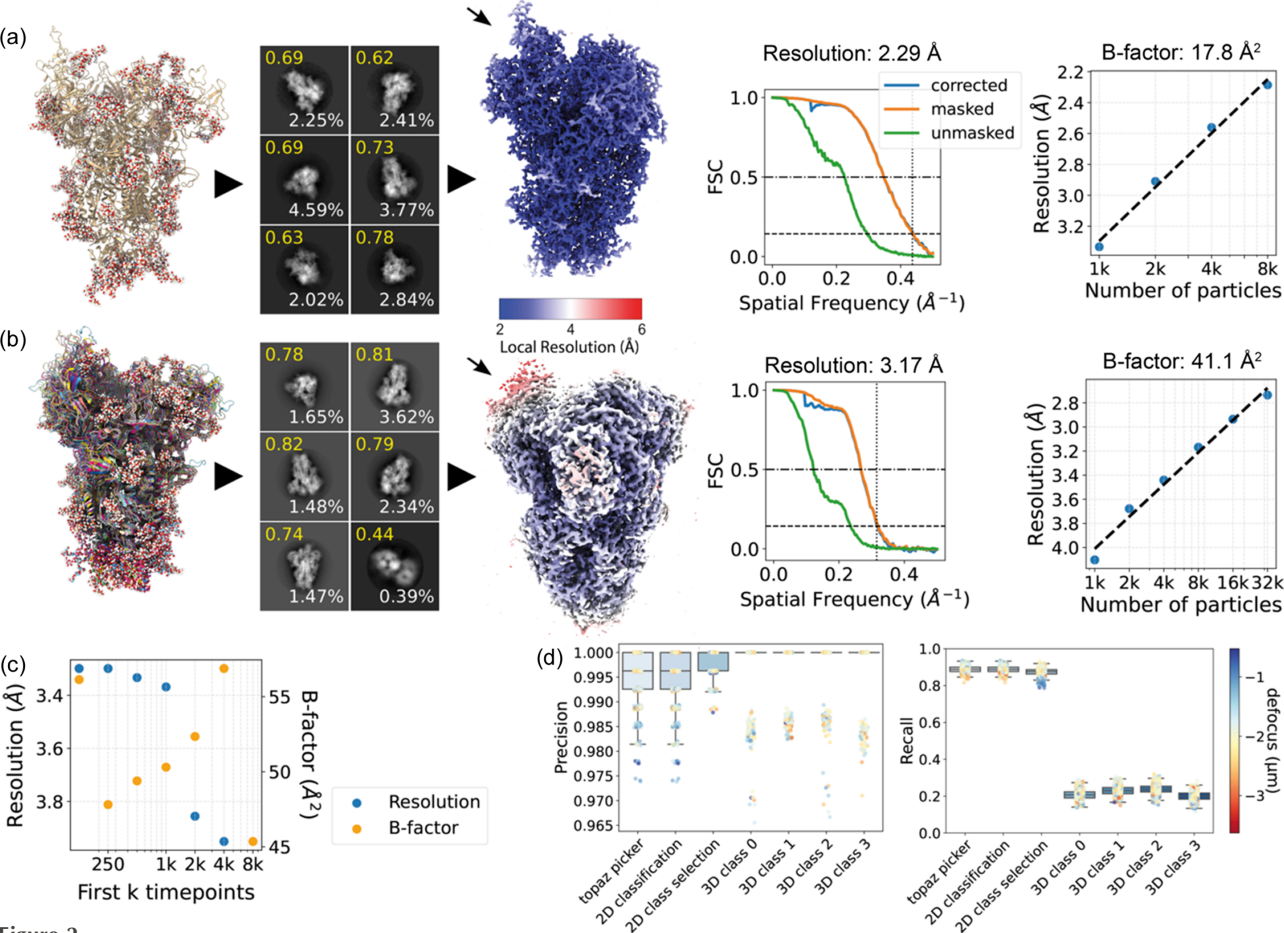
Analysis of reconstructions of single-conformation and conformationally heterogeneous datasets. (*a*) Single-conformation dataset. Atomic model (far left), 2D classes (left), 3D reconstruction (middle), FSC curve (right) and ResLog analysis (far right). For 2D classes, the ‘class score’ from automatic class ranking in *RELION* is shown in yellow and the percentage of particles in the class in white text. The density map is coloured by local resolution. The arrow indicates the RBD. (*b*) Conformationally heterogeneous dataset. Order of panels as in (*a*); the atomic models show an ensemble of structures from conformational sampling. (*c*) Resolution (blue, left side axis) and global *B* factor (orange, right side axis) versus number of included conformations in the dataset. The number of particles is constant for each dataset. (*d*) Precision and recall for various steps of the image-processing workflow for the conformationally heterogeneous dataset.

Fig. 2 ▸(*a*) shows the atomic model that was used during simulation of the first dataset. We processed this synthetic data in *RELION* (version 4.0; Kimanius *et al.*, 2021 ▸) and randomly sampled 8000 of the particles, from which we reconstructed a 2.3 Å resolution map. Except for the lack of motion correction, a standard reconstruction workflow as detailed in Section 4.3 was implemented. Local resolution estimation showed little variation except for the expected gradient towards the particle periphery [Fig. 2 ▸(*a*)], consistent with a single conformation and constant atomic displacement factor of the atomic model used for micrograph simulation. The Fourier shell correlation (FSC) curves of the final density map show the expected behaviour with a smooth fall-off to zero. The 3D density appears connected with expected features of side-chains and backbone atoms visible in the structure (see Fig. S10). Fig. 2 ▸(*a*) also shows a plot of inverse resolution versus the logarithm of the number of particles (commonly referred to as a Rosenthal–Henderson or ResLog plot (Rosenthal & Henderson, 2003 ▸; Stagg *et al.*, 2014 ▸) for this dataset where we obtained a *B* factor of 17.8 Å^2^ from the slope of a linear fit. Since the sample is perfectly homogeneous and no *B* factor is applied during simulation of these images, this overall *B* factor can be solely attributed to errors in processing such as suboptimal alignment.

The second dataset of synthetic micrographs was then pre-processed using the same workflow as the single-conformation dataset. To compare a consensus reconstruction of this dataset with a consensus reconstruction of the previous single-conformation dataset, we similarly selected 8000 particles randomly before refinement. The resulting density map is shown in Fig. 2 ▸(*b*). We now observe a lower global resolution of 3.2 Å as well as larger differences in local resolution compared with the single-conformation dataset. In the single-conformation dataset, we measured a mean local resolution of 2.2 Å with a variance of 0.61 Å^2^, while the conformationally heterogeneous dataset had a mean of 3.0 Å with a variance of 1.1 Å^2^. The largest difference in resolution is observed in the flexible receptor binding domain (RBD), which is in a partially open conformation in this MD trajectory. Performing a ResLog analysis shows the overall *B* factor has increased to 41.1 Å^2^.

We further investigated the relation between heterogeneity present in the dataset and the global resolution of a reconstructed density map. To this end we created 7 subsets of 4000 particles from the multiple-conformation dataset. Each subset only includes particles from the first *k* time points of the MD trajectory with *k* ∈ (125, 250, 500, 1000, 2000, 4000, 8000). Fig. 2 ▸(*c*) shows the global resolution and 3D refinement-estimated *B* factor as a function of *k*. As expected, the global resolution decreases and the *B* factor increases as the conformational heterogeneity contained in a subset increases. The overall resolution was lower compared with the consensus reconstructions of the single- and multiple-conformation datasets (possibly caused by the omission of the CTF refinement). The *B* factor data also show unexplained outliers which might result from the low number of particles used in refinement and the stochastic nature of the refinement.

As ground truth particle positions, orientations and CTF parameters are known, we next investigated how well the corresponding estimates from the 3D reconstruction workflow agreed with the ground truth. Fig. S1(*a*) of the supporting information plots the estimated defocus values against the ground truth defocus for each micrograph, from which we computed a correlation coefficient of 1.0 to 6 significant digits. We also compared ground truth particle orientations with estimated orientations during 3D refinement with distributions of elevation and azimuthal angles shown in Figs. S1(*b*) and S1(*c*). The ground truth distribution of viewing directions is uniform, whereas the estimated orientations show a peak which may indicate misalignment for some particles.

To analyse particle picking, we matched each picked particle with the closest truth particle within a radius of 50 Å. We define true positive picks as those picked particles which were successfully matched to a truth particle and false positives (FPs) as those which were not. From these definitions we can compute particle picking precision and recall for a set of picked and ground truth particles. Precision and recall for various steps of processing of the heterogeneous dataset are plotted in Fig. 2 ▸(*d*) on a per-micrograph basis. Particle picking is nearly perfect; a trained *Topaz* (Bepler *et al.*, 2019 ▸) model can pick 88.8% of all particles with 99.5% precision. As we will discuss in Section 2.4, we also simulated a steered MD trajectory of only a monomer of the SARS-CoV-2 spike protein. Compared with the trimer, particle picking was found to be more difficult for this smaller structure. Fig. S1(*e*) shows particle picking precision and recall for the smaller structure and here *Topaz* finds 92.3% of all particles with a precision of 82.6%. The plot also shows that, in general, the recall is higher in micrographs with a larger defocus, while the precision is lower for both the blob picker and the subsequent *Topaz* picking.

### 2.3. Radiation damage and fluence

One problem with the data simulation is the lack of frequency-dependent attenuation of the signal, which would be expected for experimental data because of beam-induced motion, radiation damage and detector response. *Parakeet* offers the option to model the detector response using a detective quantum efficiency (DQE) model which attenuates amplitudes in a frequency-dependent manner according to tabulated values based on a Falcon4 detector at 300 keV. The DQE also depends on the electron flux density; in our still image simulation, we used 5 e^−^ Å^−2^ s^−1^. In addition, to account for the effect of radiation damage on the Coulomb potential of the sample, a beam damage model is implemented as a Fourier filter, which effectively convolves the electrostatic potential with a Gaussian function whose variance is proportional to a *B* factor. This *B* factor *B* = 8π^2^*D*_E_*S*_E_ depends linearly on the fluence *D*_E_ and a sample-dependent sensitivity coefficient *S*_E_ with units Å^4^/e^−^ (Parkhurst *et al.*, 2021 ▸). In our application, the *B*-factor model serves to progressively blur the atomic potential for each subsequent frame during simulation of a dose-fractionated movie to recapitulate empirical observations of progressive beam damage in which the first few frames of a dose-fractionated movie are least affected by radiation damage and the later frames progressively lose high-frequency information (Grant & Grigorieff, 2015 ▸).

**Figure 3:**
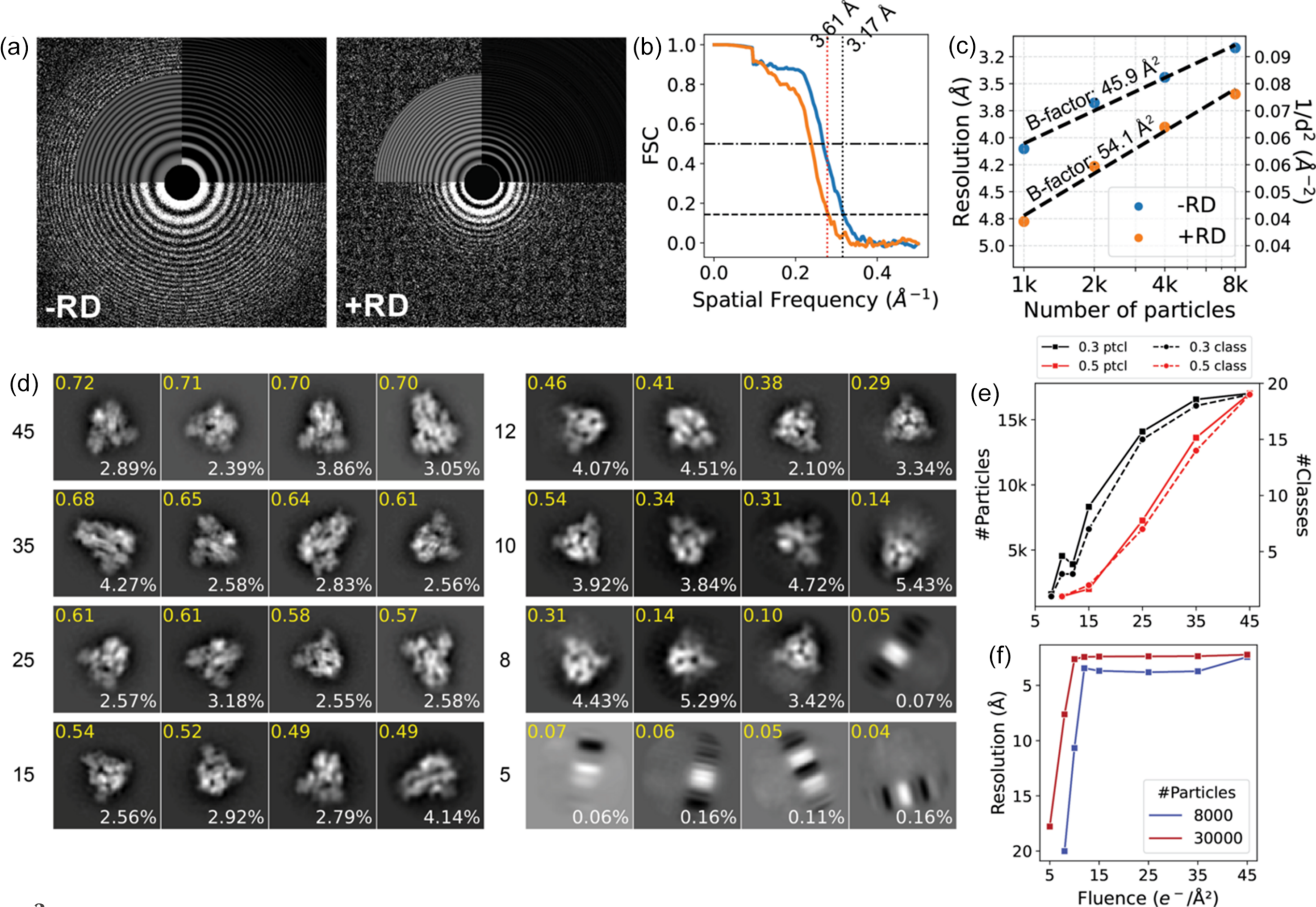
RD and fluence. (*a*) Power spectra of two micrographs simulated without (left) and with (right) RD. (*b*) FSC curves of reconstructions obtained with 8000 particles from datasets without (blue) and with (orange) RD. (*c*) ResLog analysis of datasets simulated without (blue) and with (orange) RD; the *B* factor from linear regression are shown. (*d*) 2D class averages from eight datasets with fluence ranging between 5 and 45 e^−^ Å^−2^. The four best classes are shown along with their *RELION* class ranker score in yellow and the percentage of particles in each class in white. (*e*) Number of particles (left axis, solid line) and 2D classes which pass the threshold (right axis, dashed line) of 0.3 (black) or 0.5 (red) for the predicted class scores from *RELION*. (*f*) Resolution versus fluence plots using either 30 000 (red) or 8000 (blue) particles.

To illustrate the effect of the radiation damage (RD) model, in Fig. 3 ▸(*a*) we compare exemplary power spectra from two simulated datasets. The first is from the heterogeneous SARS-CoV-2 trimer dataset introduced in Section 2.2 which contains non-fractionated micrographs without RD (−RD). The second is computed from the sum of a 30-frame dose-fractionated movie with RD enabled (+RD). In both cases, the DQE model was enabled and the total fluence was 45 e^−^ Å^−2^. Micrographs corresponding to the power spectra are shown in Figs. S2(*a*) and S2(*b*). To comparatively evaluate the effect of the radiation damage model on the 3D reconstruction, we once again computed a consensus reconstruction using 8000 randomly selected particles from the SARS-CoV-2 spike glycoprotein simulated with radiation damage, yielding a reconstruction with a global resolution of 3.6 Å as shown in Fig. S2(*c*). The FSC curve is plotted along with the corresponding FSC curve of an analogous reconstruction without the RD model [Fig. 3 ▸(*b*); see also Fig. 2 ▸(*b*)]. The ResLog analysis, shown in Fig. 3 ▸(*c*), resulted in a *B* factor of 54.1 Å^2^, compared with 45.9 Å^2^ without RD, suggesting that RD has a relatively minor effect under the conditions used in our simulations. We note that no beam-induced motion model is currently applied in either of the datasets.

In addition to RD, we also investigated the effect of fluence on various steps of the reconstruction process. We simulated a series of eight datasets with fluences of 45, 35, 25, 15, 12, 10, 8 and 5 e^−^ Å^−2^, each consisting of 100 micrographs containing 30 000 particles of the SARS-CoV-2 spike protein in total.

We picked particles from the micrographs in each dataset using the Laplacian of Gaussian (LoG) picker in *RELION*, followed by 2D classification with the relion class ranker (Kimanius *et al.*, 2021 ▸). We found that the quality score of the classes decreased with lower fluence. Fig. 3 ▸(*d*) plots the four highest scoring classes for each dataset. Visually, classes obtained from datasets with lower fluence also appear more blurred, or poorly centred. Fig. 3 ▸(*e*) plots the number of 2D classes and constituent particles retained after 2D class selection using the automated class ranker with threshold values of 0.3 or 0.5. The plot indicates that for low signal-to-noise ratio (SNR) data the main bottleneck in the reconstruction of these simulated images becomes the alignment and 2D classification. To further illustrate this, we used the ground truth particle positions to compute the average distance between each picked particle and its nearest ground truth particle location. The distribution of these distances is plotted in Fig. S2(*f*), which shows that the accuracy of the picked particle locations decreases with decreasing fluence. This may contribute to the 2D classification failing to centre the classes for low-fluence datasets.

We obtained reconstructions of each dataset using ground truth coordinates of the particles and the same reference density. The resolution of these density maps is plotted in Fig. 3 ▸(*f*) as a function of fluence. We found that the resolution remains roughly constant until the fluence drops below 10 e^−^ Å^−2^. In the absence of RD the high-frequency information in the images is over-represented compared with experimental data, causing the resolution to remain constant until the alignment fails. In all subsequent case studies, we opted to use 45 e^−^ Å^−2^.

### 2.4. Discrete classification of simulated dataset with multiple conformations

**Figure 4:**
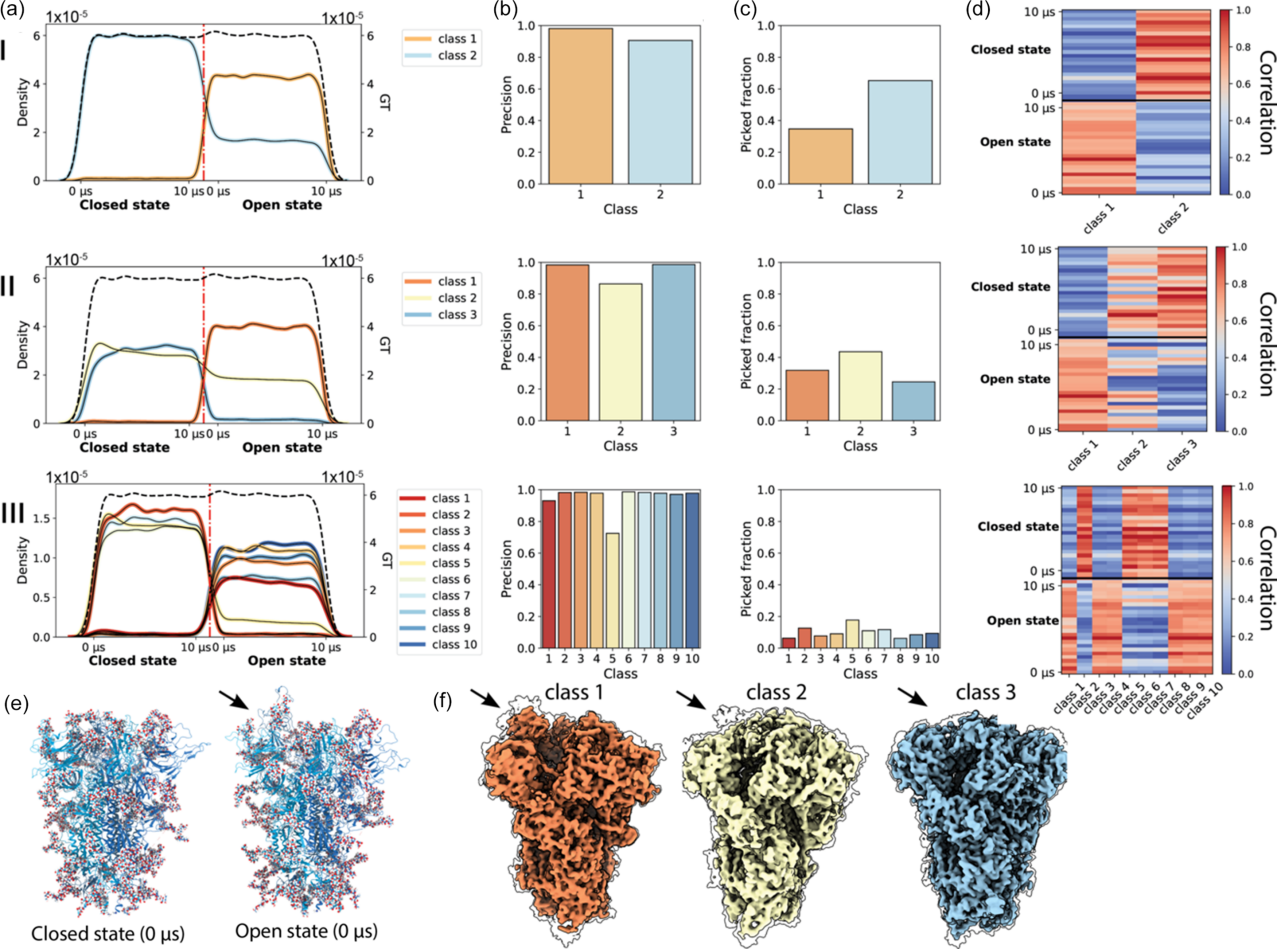
Discrete classification of conformationally heterogeneous synthetic data. (*a*) Distribution of particles in each class over the 10 µs trajectories of the spike protein in closed and open states in the case of 2 classes (I), 3 classes (II) and 10 classes (III). (*b*) Precision of each 3D class. (*c*) Fraction of particles in each class. (*d*) Correlation heat map where each class (*x* axis) is correlated against snapshots taken from the MD trajectories of the closed and open states (*y* axis). The value plotted is the real-space correlation between class and MD time point, normalized per column. (*e*) Single conformation from the closed and open states of the protein. (*f*) Three classes resulting from classification II; transparent contours outline confidence maps thresholded at 5% FDR. The arrow highlights the RBD.

Next, we applied our pipeline to analyse 3D classification of a synthetic dataset comprised of both discrete and continuous heterogeneity. For this purpose we created a dataset with two discrete structural states by mixing conformations from the previously used DESRES-ANTON-11021571 trajectory, in which the spike glycoprotein is in a partially open conformation (single RBD up), with conformations from the DESRES-ANTON-11021566 trajectory (Shaw, 2020 ▸), in which the protein is in the closed conformation (all RBDs down). We simulated 400 micrographs containing 60 081 particles from the open-state trajectory and 59 919 particles from the closed-state trajectory and processed them to obtain an initial consensus density map. From there we performed 3D classification and analysed the distribution of particles in the open and closed conformations in each 3D class. Fig. 4 ▸(*a*) shows this distribution for the case of two classes (I), three classes (II) and ten classes (III). We observed that using two classes did not result in a clean split between the trajectories, as 78.2% of particles in class two originated from the closed-state conformation and 21.8% from the open state compared with 2.1% closed state and 97.9% open state for class 1. In the case of three classes, classes 1 and 3 neatly distinguished between the open and the closed states, respectively, but class 2 was a mix of both the closed and open states (62.2% closed state, 37.8% open state). In the case of ten classes, we found that all classes contained mostly particles from one of the trajectories, with class five being the least uniform (88.3% of particles originated from the closed state).

We found that FP particles tend to accumulate in one or two classes during classification, which is also typically the class with the most particles and the most equal contribution from both trajectories. This is illustrated by Fig. 4 ▸(*b*), which shows the precision of the particle set for each 3D class, whereas Fig. 4 ▸(*c*) shows the distribution of the total number of particles over the classes. From refinement of each class of the three-class classification example, we found the resolutions to be 2.5, 2.8 and 2.6 Å, respectively. Class 2, despite containing more particles, resulted in a lower-resolution density map.

We then measured the real-space map-to-model correlation using the density map reconstructed from each class after refinement and the backbone atoms of atomic models from 50 evenly spaced time points from the MD trajectories of the closed and open states. Fig. 4 ▸(*d*) shows a heat map of the normalized correlation between each pair of atomic models and density maps. When using two classes, we found that each class has the highest correlation with atomic models from the trajectory comprising the majority of its constituent particles. When using three classes, we found that this was also true for class 1 (98.5% of particles originated from the open state and the density map correlates more strongly with atomic models of the open state) and for class 3 (95.1% of particles originated from the closed state and the density map correlates slightly better on average with atomic models in the closed state). Class 2, containing particles from both the open and the closed states, correlates strongly with atomic models in the closed state. In the case of ten classes, we again find that each class correlates strongly with atomic models from the trajectory comprising the majority of its constituent particles. Unnormalized versions of these correlation heat maps [Fig. S3(*a*)] also show that there is a large difference between classes. Visual inspection of the density maps for these 3 classes, shown in Fig. 4 ▸(*f*), shows that class 1 resembles the open state as showed by the RBD (see arrow) being in the up conformation. Although class 2 and class 3 do not show clear evidence for the RBD in the up conformation even at low-density thresholds, false discovery rate (FDR)-controlled confidence maps (Beckers *et al.*, 2019 ▸) computed for all reconstructions reveal a detectable signal for the RBD in the up conformation in classes 1 and 2, but not class 3, consistent with the class distributions determined in Fig. 4 ▸(*a*).

In addition to the synthetic dataset created by mixing the 10 µs trajectories of the molecule in the closed and partially open states, we performed a short steered MD simulation interpolating between the closed and partially open states. To reduce computational complexity we selected only one monomer of the complex out of PDB models 6xm4 (open state, RBD up) and 6xm5 (closed state, RBD down) (Zhou *et al.*, 2020 ▸). We then used harmonic restraints to drive the molecule from the open conformation to the closed conformation using openMM (Eastman *et al.*, 2017 ▸). Out of the resulting 100 ps trajectory we sampled 10 000 conformations, which were used to create 800 micrographs comprising a total of 200 000 particles. As discussed in Section 2.2, the smaller size of the particles meant preprocessing was more difficult and more particles were needed. *Ab initio* model building with 4 classes was done in *CryoSPARC* using 213 486 picked particles, of which 168 556 were true positives. Similarly to the SARS-CoV-2 spike glycoprotein trimer, we again observed 3D classes distinguishing between the open and closed conformations of the molecule. The distribution of the particles in each class over the steered MD trajectory is shown in Fig. S3(*b*). Classes 2 and 3 had substantially lower precision (0.61 and 0.62 compared with 0.99 and 0.97 for classes 0 and 1) and failed to produce initial models that resembled the spike monomer. These classes also contained particles from the entire trajectory, in contrast to classes 0 and 1 which contained mostly particles from the last 60 ps and the first 40 ps of the trajectory, respectively. All 3D classes, refined density maps and their comparison with atomic models are shown in Figs. S3(*d*) to S3(*f*).

### 2.5. Continuous heterogeneity in the latent space is well preserved

Synthetic data simulated with *Roodmus* are, by design, well suited for heterogeneous reconstruction methods that aim to derive a latent representation of the images. In this section, we showcase the application of two such methods: *CryoDRGN* (Zhong *et al.*, 2021 ▸) and *3DFlex* (Punjani & Fleet, 2023 ▸). We apply the former to a synthetic dataset of the SARS-CoV-2 spike trimer glycoprotein in its partially open conformation as well as the SARS-CoV-2 replication transcription complex (RTC) and the latter to steered MD simulations of the SARS-CoV-2 spike glycoprotein (monomer) and the protein complement C3, a component of the human complement system. These datasets differ in molecular mass, dimensions and number of particles, the magnitude of the conformational change, the timescale simulated and the coarseness with which states are sampled.

**Figure 5:**
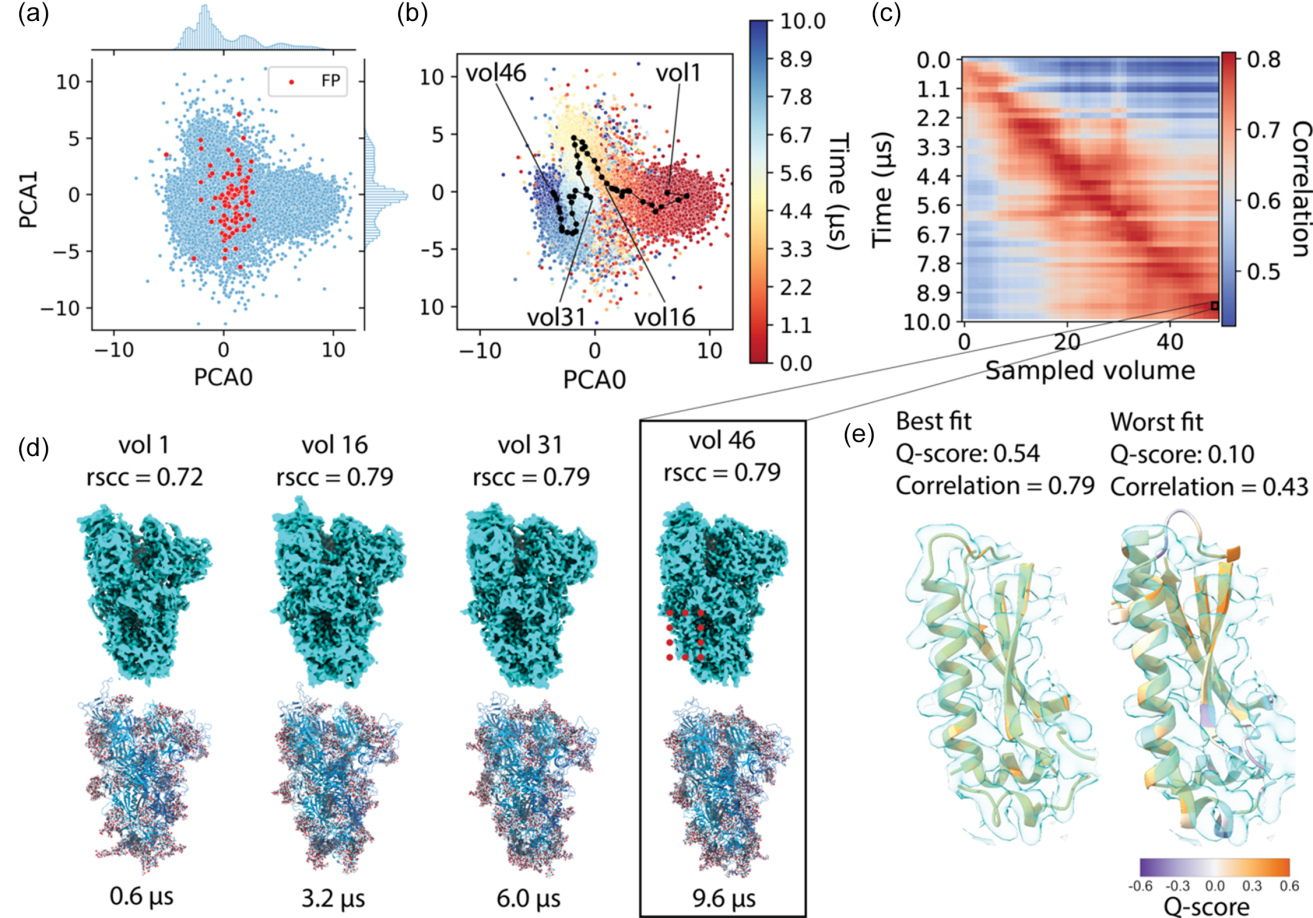
Analysis of heterogeneous reconstruction of the SARS-CoV-2 spike trimer. (*a*) Plot of the latent space after applying PCA. Red points indicate the FP particles in the latent space. (*b*) Each particle embedding is coloured according to the frame of the MD trajectory the particle originated from. Black dots show a trajectory traced through the latent space. (*c*) Real-space correlation between 50 volumes generated by sampling the latent space (*x* axis) and frames in the MD trajectory (*y* axis). (*d*) Example volumes sampled from the latent space with the frame of the trajectory for which it showed the highest correlation. (*e*) Zoom of the density map of volume 46 with the best and worst atomic models out of the 50 sampled states from the MD trajectory displayed. Atomic models are coloured by backbone Q-scores.

The open-state SARS-CoV-2 dataset is the same as the conformationally heterogeneous dataset used for the consensus reconstructions in Section 2.2. The final cleaned data consist of 236 079 particles taken after performing a 3D refinement in *RELION*. The default 8-dimensional latent space was used for *CryoDRGN* training. In Fig. 5 ▸(*a*) we show a 2D embedding of the latent space using PCA for dimensionality reduction. FP particle picks, which make up 0.03% of the dataset, are coloured red and are spread throughout the latent space. Fig. S4(*a*) shows a density plot that visualizes how the latent space embeddings do not form distinct clusters. We then use ground truth information to colour each particle according to the time point of the MD trajectory from which it originated, as shown in Fig. 5 ▸(*b*). Conformations that are close together in time were also found to cluster together in the latent space embedding. We grouped the MD trajectory into 50 batches of conformations, each representing a contiguous 2% of the total number of time points sampled in the ground truth MD trajectory. By calculating the mean latent coordinates of particles in the latent space which originated from each batch, the average path of the MD trajectory through the latent space embedding was traced. The generated path progresses continuously through the latent space embedding. We then used the trained decoder to evaluate these selected latent coordinates and produce their corresponding 3D density maps.

To evaluate the decoding of the latent space into density maps, we compared the 50 generated maps with 50 atomic models at equidistant time intervals in the MD trajectory by real-space map-to-model correlation and plotted those in a 2D heat map shown in Fig. 5 ▸(*c*). The heat map features a strong diagonal, indicating that the sampled volumes are most similar to the conformation of the molecule in the particles around the selected latent coordinate. We highlight a few sampled density maps together with the atomic model which has the highest correlation to the map in Fig. 5 ▸(*d*). A more detailed map-to-model fit is shown in Fig. 5 ▸(*e*) where volume 46 is shown with a close-up of the best and worst fitting atomic models. We further validated the map-to-model fit by calculating the average Q-scores (Pintilie *et al.*, 2020 ▸) for both models and found a Q-score of 0.54 for the best fitting model compared with a score of 0.10 for the worst fitting model. Atomic structures are coloured according to backbone per-residue Q-score. Entire atomic models coloured by the backbone per-residue Q-score are shown in Figs. S4(*b*) and S4(*c*).

In addition, we trained *CryoDRGN* on a similar dataset where RD was enabled, as described in Section 2.3. The latent space is plotted in Fig. S5. We again found that *CryoDRGN* was able to organize the particles according to their time point in the MD trajectory, despite the decreased SNR of the high-frequency information and the comparatively small number of particles (15 846) used to train *CryoDRGN* in this case.

**Figure 6:**
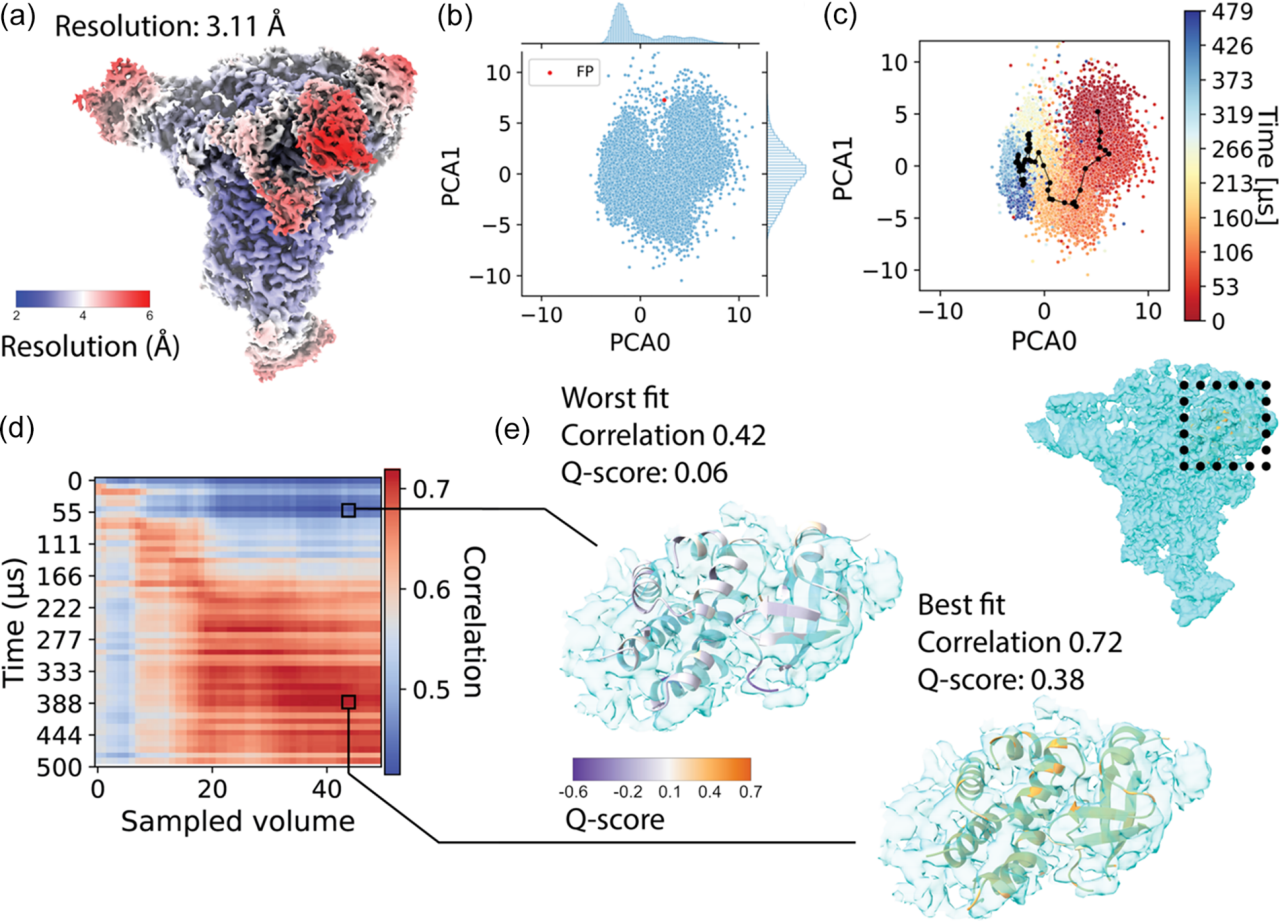
Analysis of heterogeneous reconstruction of the SARS-CoV-2 RTC. (*a*) Consensus reconstruction of the RTC, coloured by local resolution. (*b*) Plot of the latent space after applying PCA. (*c*) Same latent space, coloured according to the frame of the MD trajectory the particle originated from. (*d*) Real-space correlation between 50 volumes generated by sampling the latent space (*x* axis) and frames in the MD trajectory (*y* axis). (*e*) Best and worst atomic models shown in the density map of volume 43.

Next, we repeated the same steps for a second dataset based on the SARS-CoV-2 RTC. The MD simulation that was used as a source of heterogeneity for this synthetic dataset is much longer (500 µs compared with 10 µs for the spike protein) and sampled with a time interval of 4.8 ns (Shaw, 2020 ▸). From this trajectory, 10 000 frames were sampled and 167 micrographs were simulated with a total of 50 100 particles. Fig. 6 ▸(*a*) shows the consensus reconstruction obtained with 41 146 particles, which reached 3.11 Å global resolution. Despite the much longer simulation, *CryoDRGN* again was able to organize the latent space coherently with time points in the MD trajectory from which the particles were sampled, as exemplified in Figs. 6 ▸(*b*) and 6 ▸(*c*). We used the same approach as for the open-state SARS-CoV-2 dataset to compute a real-space correlation matrix, which is shown in Fig. 6 ▸(*d*). Good agreement was found between the time points in the MD trajectory and the generated volumes, although towards the later time points in the trajectory we see broader correlations with the volumes generated. This may indicate that conformations become less distinguishable. This conclusion is supported by the averaged path through the latent space in Fig. 6 ▸(*c*), and the lack of separation in the latent space between later time points in the MD simulation is easily observed in the high-density region in Fig. S4(*d*). Finally, we show two examples of a small section of the atomic model in a flexible region of the complex in Fig. 6 ▸(*e*); the best and worst fit according to real-space map-to-model correlation. The entire atomic model coloured by backbone Q-scores is shown in Figs. S4(*e*) and S4(*f*).

As discussed in Section 2.2 we also performed a steered MD simulation of the SARS-CoV-2 spike protein. We reduced the complex to a single monomer and forced a conformational change between an open and closed state of the RBD using harmonic restraints. We then trained *CryoSPARC*’s *3DFlex* model using 1D and 2D latent spaces. The latent spaces of both trained models are shown in Figs. S6(*a*)–S6(*d*) (for the 1D latent space we show the distribution of latent coordinates). In the case of a 1D latent space we find that there is some correlation between the estimated latent encoding and the time point in the MD trajectory, whereas in the 2D latent space we find that the latent space is not organized in a continuous manner. Unlike our previous examples, there is no path through the cluster that can reconstruct the MD trajectory in chronological order. Correlation between the time points in the MD trajectory and the sampled volumes, shown in Figs. S6(*e*) and S6(*f*), show a strong relation between conformations in the MD trajectory and the sampled volumes in the case of a 1D latent space, but not for a 2D latent space.

We tested *3DFlex* with another synthetic dataset based on a steered MD simulation of the complement system protein C3, based on a morph trajectory interpolating between PDB entry 2a73 (Janssen *et al.*, 2005 ▸) and PDB entry 2i07 (Janssen *et al.*, 2006 ▸). C3 undergoes a major conformational change when transitioning from its inactive state C3 to the active C3b that exposes a reactive thio­ester for opsonization of target surfaces (Janssen *et al.*, 2006 ▸). A visualization of the ensemble of states generated during the steered MD simulation is shown in Fig. S7(*a*) and a consensus reconstruction from 96 169 particles with 2.55 Å global resolution in Fig. S7(*b*). We then trained a *3DFlex* model with a 2D latent space on these data and obtained a latent space where different sections of the MD trajectory were clustered together. Due to the magnitude of the conformational change, we ran the steered MD simulation in four iterations with the target state first set to a conformation a third of the way between the initial and final states, then being changed to a state two-thirds of the way and then being changed to the final state for the last two iterations. As a result, we expected that the latent space might be split into three discrete clusters that resemble the target states. We observe three regions in the latent embedding corresponding to the first 45 ps, the next 45–90 ps and the last 110 ps time points. However, there is not a continuous path through the latent space following the chronological order of the trajectory and these regions are not well separated.

## 3. Discussion

Many biomolecules have evolved to perform specific tasks through a concerted sequence of conformational motions. The current trend in SPA is to address conformational heterogeneity with machine-learning algorithms in order to identify and sort a continuum of structural states into a continuous representation of heterogeneity which may be correlated to functional motion. Here we have demonstrated a new toolkit for the simulation of single-particle cryo-EM micrographs that contain conformational heterogeneity, investigated the effect of this heterogeneity on consensus reconstructions and explored the ability of two established machine-learning-based HRAs to quantitatively recover the ground truth conformational heterogeneity from a collection of single-particle snapshots.

We found that training both *CryoDRGN* and *3DFlex* resulted in models for which particles with conformations that were temporally close in the MD trajectory were also clustered closely together in the latent space. Using *CryoDRGN* to process the SARS-CoV-2 spike glycoprotein and RTC synthetic datasets we found that it was possible to generate a path through the latent space such that the reconstructed volumes correlated strongly with evenly spaced time points of the MD trajectory.

The ultimate goal would be to decode the chronological order of states underlying functionally relevant conformational trajectories. However, at present we focus on structures that are conformationally similar during traversal through latent space despite the non-linear embedding. One area of future study could be devoted to developing and evaluating methods to interpret these learned latent spaces. One such example is *CryoDRGN* conformational landscape analysis (Zhong, 2022 ▸) which, through analysis in volume space, both clusters a small number of discrete conformational states and allows inference of continuous reaction coordinates. With the presence of ground truth information, investigating the optimization of dimensionality reduction and clustering methods applied to both MD trajectories and latent spaces may provide insights on the interpretation of both.

Our analysis also highlights that more work is needed to understand the requirements on data quality for HRAs to recover physically relevant conformational trajectories. In this paper, we limited our investigation to a coarse analysis of the effects of RD and fluence on conventional reconstructions, but it is possible to conduct systematic studies into the behaviour of HRAs in the limit of poor data quality.

Other aspects of cryo-EM image processing workflows can also be investigated using this approach. We have demonstrated this for particle picking using the ground truth particle positions to compute precision and recall for a set of picked particles. As this aspect of the pipeline often involves parameter optimization as well, it may be beneficial to investigate the behaviour of particle pickers, 2D/3D classification algorithms and refinement algorithms in the limit of strong conformational or compositional heterogeneity, very poor SNR, unevenly sampled particle orientations or poor annotations of training data. It is also possible to simulate experimental pathologies such as orientation bias and to investigate the effect this has on conventional 3D reconstruction workflows or HRAs. Preliminary analysis with the SARS-CoV-2 spike trimer suggests that the effect of orientation bias on the ability of HRAs to sort the latent space according to the ground truth trajectory is complex and that in such cases latent spaces need to be interpreted with caution. Further in-depth studies, including the consequences of point-group symmetry, are warranted to thoroughly evaluate the effect of orientation bias on the performance of HRAs. Conversely, *Roodmus* also makes it possible to study the effects which future developments in instrumentation and algorithms may have on the ability to reconstruct heterogeneity.

*Roodmus* sources conformational heterogeneity from MD simulations. This presents a major advantage over previous related methods where the conformational heterogeneity was generated by interpolating between two or more discrete structural states. Atomistic ensembles from MD simulations allow evaluation of HRAs in more physically realistic settings but come with the disadvantage that large-scale domain motion is computationally expensive to simulate at the atomistic level. Coarse-grained MD or enhanced sampling strategies may be a solution that could allow for more in-depth exploration of the behaviour of both conventional reconstruction workflows and HRAs in the case of large-scale motion, or in cases where intermediate states are less densely populated.

Image simulation with accurate modelling of experimental image contrast and optical aberrations is essential to evaluate HRA performance on realistic cryo-EM datasets with independent ground truth information. The multislice forward model of the *Parakeet* software offers improved cryo-EM image simulation methods compared with alternative ways of generating synthetic data. An electron wave is propagated through a virtual sample, as opposed to a simple linear projection through the volume. This more accurately simulates the projection of the specimen’s electrostatic potential. *Parakeet* included models for detector response, contrast modulation by the CTF and a sophisticated model for structural noise contributions from an amorphous ice layer. We found no qualitative effect of *Parakeet*’s RD model on the training of *CryoDRGN* and we still obtained consensus reconstructions with unusually low *B* factors compared with the experimental data. This suggests refinement of the model is required to more accurately reflect the structural damage inflicted on the specimen during electron exposure, which represents one of the main limiting factors of cryo-EM imaging (Hayward & Glaeser, 1979 ▸). An additional missing element in *Parakeet* simulations is that of beam-induced motion. Since such motion can only be imperfectly corrected for, including these effects would likely yield overall *B* factors and concomitant attenuation of high-frequency signal more similar to those observed in experimental data. Synthetic datasets that feature these properties may be more realistic starting points for investigating the data quality requirements of HRAs.

As current HRA methods evolve and improve, periodic community evaluation of these methods against ground truth data using the *Roodmus* toolkit would help quantify the performance of these methods and encourage wider usage in the field. Progress is being made in this area, with HRA benchmarking studies by Dsouza *et al.* (2023 ▸) and more recently by Jeon *et al.* (2024 ▸). Further comparisons could also be made to orthogonal experimental data for the same (or similar) biological systems. Comparison of simulated and experimental datasets may help us to understand the degree to which MD trajectories underestimate experimental disorder given their simulation time.

*Roodmus* (Greer *et al.*, 2024 ▸) is available as open-source software (https://github.com/ccpem/roodmus) and from *PyPI* (https://pypi.org/project/roodmus). The *Roodmus* toolkit provides the necessary modular utilities to generate synthetic SPA datasets and explore the performance of processing algorithms on them. It is important to be aware that *Roodmus* can only be used to explore heterogeneity within the bounds of the MD simulation data provided to it, and that there are avenues for improvement in the simulation protocol; however, we believe that *Roodmus* facilitates the undertaking of a range of studies for elucidation of conformational heterogeneity investigation techniques. We have reported findings from studies utilizing both discrete and continuous approaches to explore heterogeneity on a number of synthetic datasets but there is ample opportunity for further work to better understand conventional reconstruction pipelines, the data quality requirements of HRAs, and the traversal and clustering of latent encodings of heterogeneity. There is also scope to investigate the relation between synthetic and experimental SPA datasets, methods for improvement of synthetic micrograph generation and the extension of *Roodmus* to tomography.

## 4. Methods

### 4.1. MD trajectories

Publicly available MD trajectories of the SARS-CoV-2 RTC and the spike glycoprotein in closed and partially open states were performed by Shaw (2020 ▸) and downloaded from https://www.deshawresearch.com. Specific trajectories were DESRES-ANTON-13795965 for the RTC, and DESRES-ANTON-[11021566,11021571] for the spike protein in the closed and partially open states, respectively.

Additional steered MD trajectories were produced using the openMM (Eastman *et al.*, 2017 ▸) Python library. PDB models 6xm4 and 6xm5 (Zhou *et al.*, 2020 ▸) were used as starting and target conformations, respectively. Chain B was isolated from both models and prepared for simulation using the pdbfixer library provided by openMM. Harmonic restraints were added between each pair of Cα atoms in the starting and target models. The system was calibrated at 0 K for 50 ps and then annealed to 300 K for another 50 ps. The force constant for all restraints was annealed between 100 and 400 ps from 0 to 10 kJ nm^−1^ mol^−1^. The integration time step was 2 fs, and the time interval between saving frames was 10 fs. After completion of the simulation, the trajectory was downsampled to 10 000 contiguous frames between 100 and 200 ps in which the molecule underwent the conformational change.

A steered MD simulation was performed on human complement component C3 (PDB entry 2a73) as it undergoes a conformational transition into a state termed C3b (PDB entry 2i07) after proteolytic activation (Janssen *et al.*, 2006 ▸). A morphing trajectory interpolating between both states was kindly provided by F. Forneris (University of Pavia). Starting from frame 1 of the morph and taking frame 31 as a target, similar harmonic restraints as described above were added between each pair of Cα atoms in the structures. An integration time step of 2 fs was again used with a time interval between saved frames of 100 fs. The system was initialized at 300 K with a force constant of 5 kJ nm^−1^ mol^−1^, which was annealed to 15 kJ nm^−1^ mol^−1^ over 200 ps. After 200 ps, the target structure was replaced with frame 61 of the morph and the force constant was annealed again from 5 to 15 kJ nm^−1^ mol^−1^ over 200 ps. Another 400 ps were simulated with frame 91 as the target state, first 200 ps with a force constant increasing from 10 to 60 kJ nm^−1^ mol^−1^, then another 200 ps with a force constant increasing from 60 to 120 kJ nm^−1^ mol^−1^.

### 4.2. Synthetic micrograph and movie simulation

Image simulation was performed using the *Parakeet* software git commit 17a0c864f6cfd84b5fd56b60fa446f7b021d338c available from https://github.com/rosalindfranklininstitute/parakeet. The *Roodmus*conformations sampling utility was used to sample a number of conformations from each MD trajectory, as indicated in Table 1 ▸. The *Roodmus*run parakeet utility was used to configure and run *Parakeet* to produce micrographs containing 300 particles (SARS-CoV-2 spike trimer and RTC) or 250 particles (steered MD datasets).

The RTC dataset was generated as movies with three frames with no particle motion between frames and no RD simulation. The movies with beam damage enabled, based on MD trajectory DESRES-ANTON-11021571 introduced in Section 2.3, were generated with 30 frames and a total fluence of 45 e^−^ Å^−2^, allowing a sensible use of the *Parakeet* RD model (Parkhurst *et al.*, 2021 ▸).

All datasets except those noted in Section 2.3 were simulated at 45 e^−^ Å^−2^, with a pixel size of 1.0 Å and an ice thickness of 50 nm which are typical for many single-particle datasets (Noble *et al.*, 2018 ▸). For lower-fluence datasets, 100 micrographs were simulated with 30 000 total particles per condition. Fluence parameters were varied between 45 and 5 e^−^ Å^−2^. Simulation of datasets was benchmarked utilizing an Intel Xeon Gold 5218 processor with 75 GB of RAM and an Nvidia Tesla V100 (32 GB) GPU. Generating a single non-fractionated micrograph following the simulation parameters used for the spike trimer (open) dataset took 5 min. Generating a single 30-frame fractionated movie with RD took 22 min. We note that the simulation time depends on a number of simulation parameters, including the number of particles, fractionation level, sample size and the physical effects being simulated.

### 4.3. Cryo-EM processing in *RELION*

The datasets created from the DESRES-ANTON-13795965 and DESRES-ANTON-[11021566,11021571] MD simulations were reconstructed using *RELION* (version 4.0). Altogether these constitute the single-micrograph datasets for DESRES-ANTON-[11021566,11021571], the DESRES-ANTON-11021571 dataset utilizing a single conformation, the 30-frame fraction­ated movie dataset for DESRES-ANTON-11021571, the 3-frame movie dataset for DESRES-ANTON-13795965 and the DESRES-ANTON-[11021566,11021571] mixed data­set. The workflows for consensus reconstructions of these are illustrated in Figs. S8, S9, S10 and S11 along with visualizations of several stages of the processing. Depicted micrographs were normalized with the ccpem-pipeliner available at https://gitlab.com/ccpem/ccpem-pipeliner.

Before CTF estimation using *CTFFIND4* (Rohou & Grigorieff, 2015 ▸), the movie datasets were motion-corrected – an unnecessary step for the single-micrograph datasets. *Topaz* (Bepler *et al.*, 2019 ▸) was trained on a manually picked subset of particles and used for autopicking. A total of 100 2D classes were generated and those with a *RELION* class ranker score greater than 0.25 were kept. After initial model building, four 3D classes were deduced, and poor classes were removed after manual inspection. Those remaining were 3D refined to produce a single consensus map suitable as the input for a ‘PostProcess’ job (to determine the global resolution after applying a mask) and for local resolution determination via a ‘LocalRes’ job. The number of particles kept at each stage of reconstruction is reported in Figs. S8 to S11.

### 4.4. Cryo-EM processing in *CryoSPARC*

The SARS-CoV-2 spike monomer steered MD dataset and the C3–C3b steered monomer datasets were reconstructed using *CryoSPARC* (version 4.2.1). For the monomeric SARS-CoV-2 spike dataset 800 micrographs were imported. CTF estimation was performed using *CTFFIND4*, followed by automatic particle picking with the blob picker algorithm, with minimum and maximum diameters of 50 and 200 Å, resulting in 1 968 039 picked particle locations. Picked particles were filtered based on the local power, extracted and 2D classified into 50 classes. A selection of 19 classes (93 574 particles) was then used for *Topaz* training, resulting in 368 967 particle picks. These picked particles were again filtered, extracted and 2D classified into 50 classes. All classes (213 486 particles) were selected for *ab initio* model building with 4 classes. The first class was selected as a reference for homogeneous refinement with all 213 486 particles. This consensus reconstruction was then used to train *3DFlex* as described in Section 4.6. The workflows for consensus reconstruction of these datasets are illustrated in Figs. S12 and S13 along with visualization of several stages of the processing.

For the C3–C3b steered MD dataset 800 micrographs were imported. CTF estimation was performed using the Patch CTF estimation job, followed by manual picking of 267 particles in 4 micrographs. These picked particles were used to train *Topaz*, resulting in 183 092 picked particle locations. The picked particle locations were filtered, extracted and 2D classified into 50 classes. All particles were kept, resulting in 96 169 particles used for *ab initio* model building with 4 classes. The second class was used as a reference for homogeneous refinement with all particles. This consensus reconstruction was then used to train *3DFlex* as described in Section 4.6.

### 4.5. Training *CryoDRGN*

Pose and CTF information obtained from the consensus reconstruction in *RELION* were extracted using *CryoDRGN* (version 3.0.0b0) according to the *CryoDRGN* tutorial (https://ez-lab.gitbook.io/cryodrgn). The model was then trained for 25 epochs with default residual MLP architecture for both the encoder and the decoder using particle images of 320 × 320 pixels (1.0 Å pixel size). The dimensionality of the latent space was 8 in all cases. The number of particles used for training was 41 146 for the SARS-CoV-2 RTC dataset (DESRES-ANTON-13795965), 236 079 for the SARS-CoV-2 spike glycoprotein dataset (DESRES-ANTON-11021571) and 15 846 for the dose-fractionated dataset.

### 4.6. Training *3DFlex*

Training the *3DFlex* model was done according to the tutorial provided by *CryoSPARC*. Particles taken from the homogeneous refinement were used for a ‘3D Flex Data Prep’ job with a training box size of 128 pixels. A mesh was created with a base number of tetrahedral cells of 30. The model was trained with a latent dimensionality of 2, 64 hidden units in the flow generator network for 24 epochs. Custom trajectories through the latent space were created with the *CryoSPARC*-tools Python library.

## Supplementary Material

Supporting figures. DOI: 10.1107/S2052252524009321/rq5011sup1.pdf

Simulated micrographs for DESRES-Trajectory_sarscov2-11021571-all-glueCA_single_conformation: https://doi.org/10.4121/e338f9af-049a-45c9-bbf9-0cd1839489ce

Simulated micrographs for DESRES-Trajectory_sarscov2-11021571-all-glueCA: https://doi.org/10.4121/b28e84c9-7959-4299-9a69-279b10250257

Simulated micrographs for DESRES-Trajectory_sarscov2-11021571-all-glueCA_single_fractionated: https://doi.org/10.4121/fe5e7ef0-b6a5-49a4-a2cd-4ecb53edf83f

Simulated micrographs for DESRES-Trajectory_sarscov2-13795965-no-water-movies: https://doi.org/10.4121/137d1031-c1ee-40d5-9a36-fd4f4b8af554

Simulated micrographs for DESRES-Trajectory_sarscov2-11021566-11021571-mixed: https://doi.org/10.4121/27ec37ba-2e8b-4c0d-ac9d-480bfb067d0d

Simulated micrographs for 6xm5_steered: https://doi.org/10.4121/eefcc341-250c-407c-a299-a4512df5f962

Simulated micrographs for c3c3b: https://doi.org/10.4121/2702bc54-def0-4daf-8cd0-7f8e85453616

Image processing results and intermediates from RELION and CryoSPARC for all datasets: https://doi.org/10.4121/55e06cd2-43cf-40d4-b2ce-ace9ce92d536

## Figures and Tables

**Table 1:** Specification of simulated datasets and refinement statistics

	SARS-CoV-2 spike trimer (closed)	SARS-CoV-2 spike trimer (open)	SARS-CoV-2 spike trimer (mixed)	SARS-CoV-2 RTC	SARS-CoV-2 spike monomer	C3-C3b
Trajectories	DESRES-ANTON-11021566	DESRES-ANTON-11021571	DESRES-ANTON-[11021566,11021571]	DESRES-ANTON-13795965	6xm4–6xm5 steered	2a73-2i07 steered
No. of conformations	8334	8334	8334 (×2)	10000	10000	2000
No. of particles	270000	270000	120000	50100	200000	200000
Acceleration voltage (kV)	300	300	300	300	300	300
Fluence (e^−^ Å^−2^)	45	45	45	45	45	45
Flux density (e^−^ pixel^−1^ s^−1^)	5	5	5	5	5	5
Pixel size (Å)	1.0	1.0	1.0	1.0	1.0	1.0
Defocus range (µm)	0.5–3.5	0.5–3.5	0.5–2.5	0.5–3.5	0.5–2.5	0.5–2.5
Refinement software	*RELION* (version 4.0)	*RELION* (version 4.0)	*RELION* (version 4.0)	*RELION* (version 4.0)	*CryoSPARC* (version 4.2.1)	*CryoSPARC* (version 4.2.1)

## Data Availability

Synthetic micrographs and atomic structure models used in data generation, as well as reconstructed density maps and image processing intermediate results, are available from the 4TU.ResearchData repository. DESRES-Trajectory_sarscov2-11021571-all-glueCA_single_conformation dataset: https://doi.org/10.4121/e338f9af-049a-45c9-bbf9-0cd1839489ce. DESRES-Trajectory_sarscov2-11021571-all-glueCA: https://doi.org/10.4121/b28e84c9-7959-4299-9a69-279b10250257. DES­RES-Trajectory_sarscov2-11021571-all-glueCA_fractionated:https://doi.org/10.4121/fe5e7ef0-b6a5-49a4-a2cd-4ecb53edf83f. DESRES-Trajectory_sarscov2-11021566-11021571-mixed: https://doi.org/10.4121/27ec37ba-2e8b-4c0d-ac9d-480bfb067d0d. DESRES-Trajectory_sarscov2-13795965-no-water-movies:https://doi.org/10.4121/137d1031-c1ee-40d5-9a36-fd4f4b8af554. 6xm5_steered: https://doi.org/10.4121/eefcc341-250c-407c-a299-a4512df5f962. c3c3b: https://doi.org/10.4121/2702bc54-def0-4daf-8cd0-7f8e85453616. Image processing results and intermediates from *RELION* and *CryoSPARC* can be downloaded for all datasets from https://doi.org/10.4121/55e06cd2-43cf-40d4-b2ce-ace9ce92d536.
