# Supplementary material for: Roodmus: a toolkit for benchmarking heterogeneous electron cryo-microscopy reconstructions
Source: IUCrJ. 2024 Oct 15;11(Pt 6):951–65. doi: 10.1107/S2052252524009321 (PMC11533995; doi:10.1107/S2052252524009321)
Supplement: Supplementary file 1 [file m-11-00951-sup1.pdf]

IUCrJ

Volume 11 (2024)

Supporting information for article:

Roodmus: A toolkit for benchmarking heterogeneous electron cryo-microscopy reconstructions

Maarten Joosten, Joel Greer, James Parkhurst, Tom Burnley and Arjen J. Jakobi

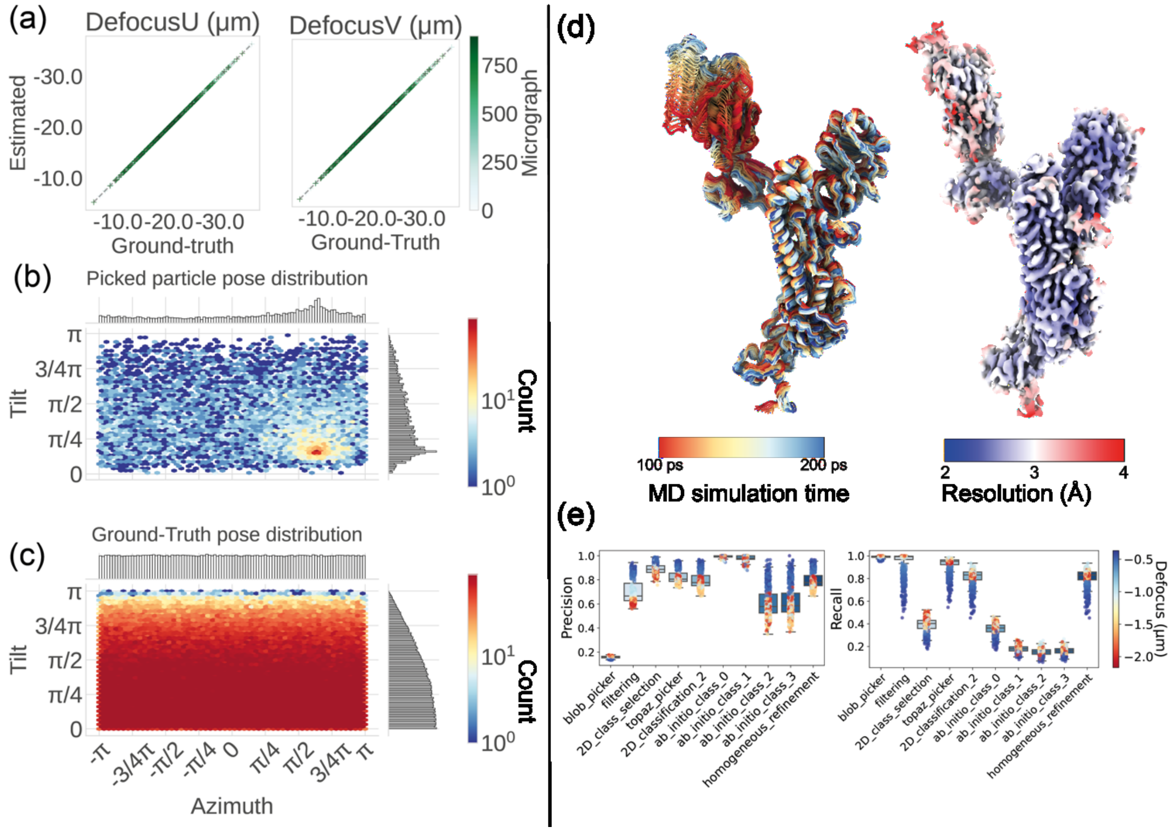

Figure S1. Further processing statistics for spike trimer protein and reconstruction of spike monomer. (a) Correlation between estimated and true defocus values for each micrograph in heterogeneous spike trimer (open) dataset. (b) Distribution of orientations estimated during 3D refinement of heterogeneous spike trimer (open) 8000 particle subset. (c) True orientations for each particle in total spike trimer (open) dataset. (d) Monomer SARS-CoV-2 spike protein, model and reconstructed consensus density map, colour indicates local resolution. (e) Particle picking precision and recall for spike monomer. X-axis indicates job types used during processing in CryoSPARC.

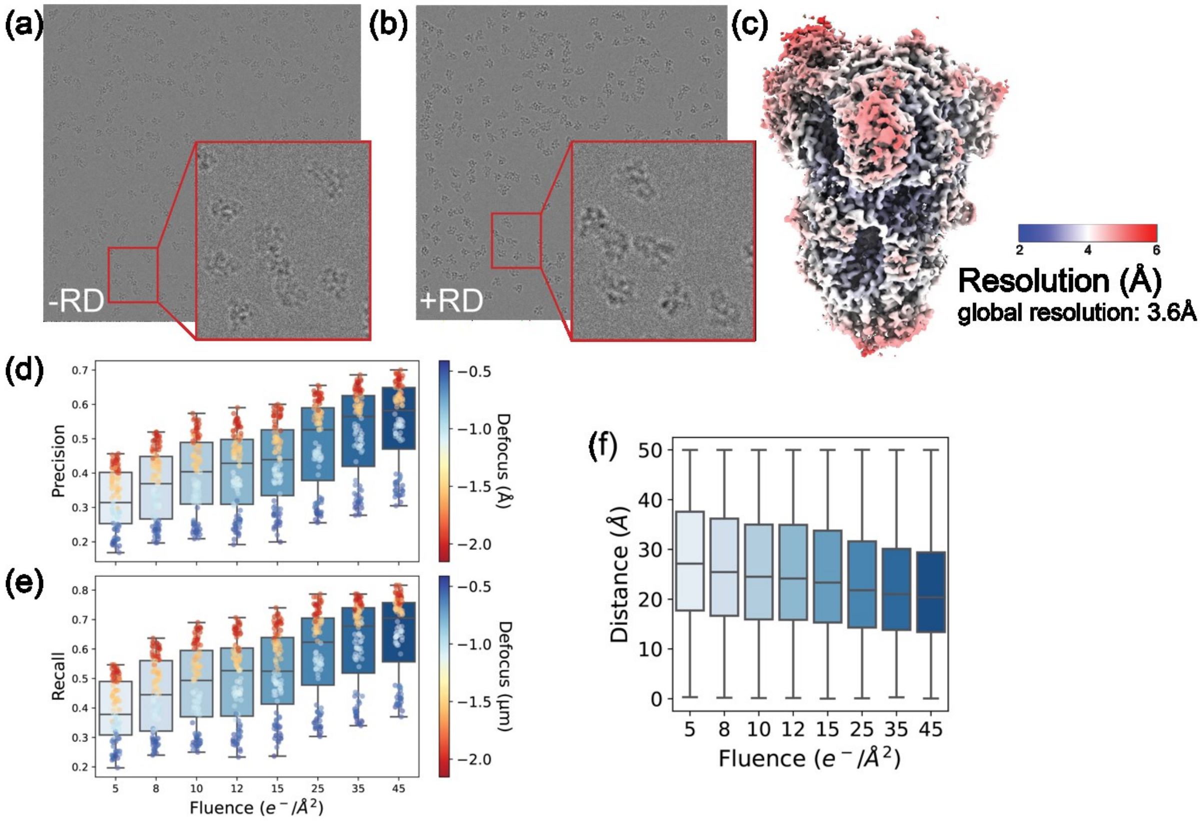

Figure S2. Effect of defocus and fluence on synthetic micrographs. (a,b) Example micrograph shown after simulation without (a) and with (b) radiation damage. Normalisation for visualisation done with the ccp-em pipeliner. (c) Reconstructed consensus density map of the dataset with radiation damage, using 15846 particles. (d,e): Picked particle precision and recall for datasets simulated with increasing total fluence. (f) Boxplot showing the distribution of distances between each picked particle and the closest ground-truth particle within 50 Å. The median decreases as fluence increases, indicating that the picked position becomes more accurate w.r.t. the ground-truth position as the fluence increases.

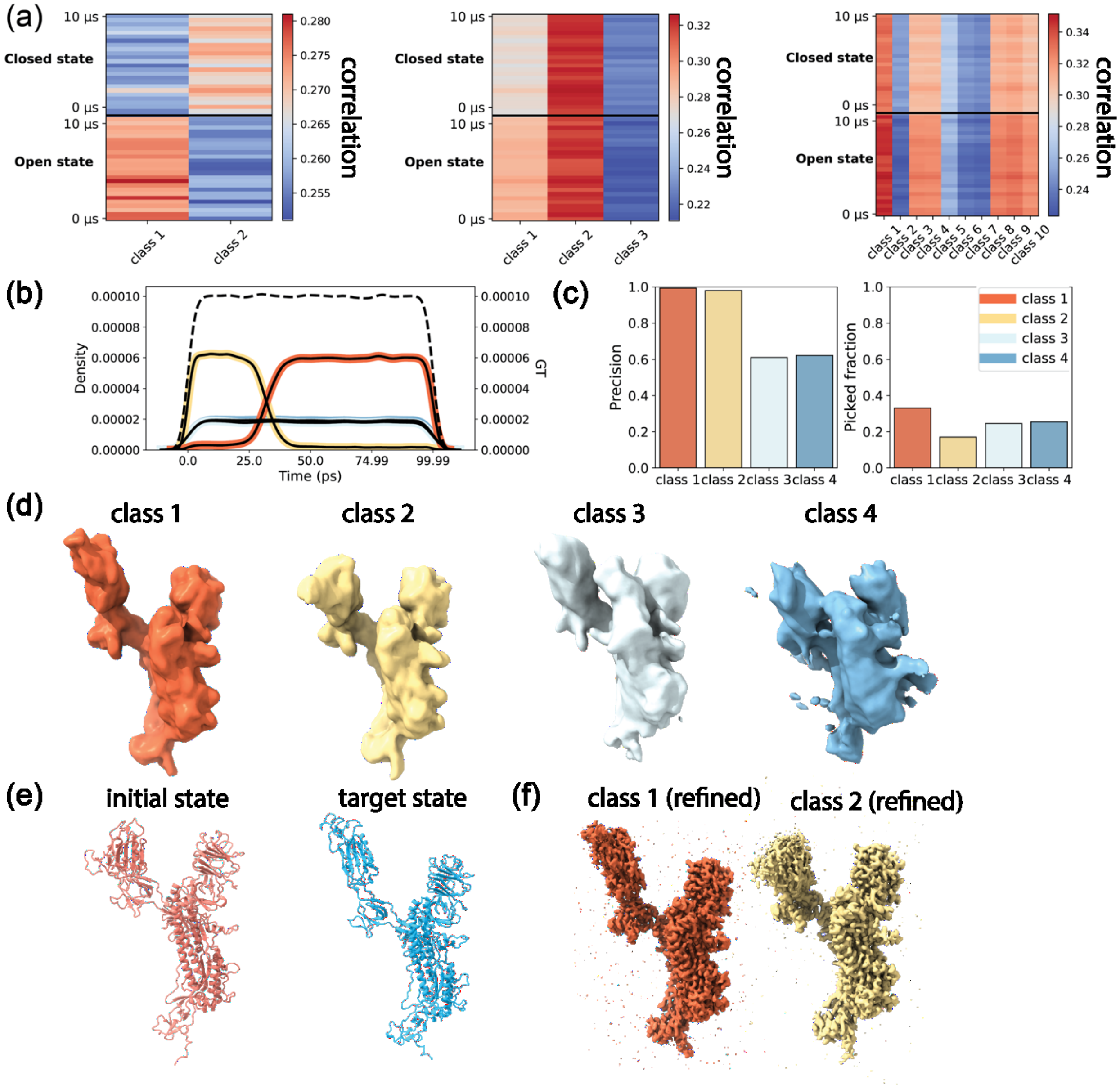

Figure S3. 3D classification of steered MD dataset of SARS-CoV-2 spike monomer. (a) Unnormalised correlation heatmaps for the 2, 3 and 10-class classification of the SARS-CoV-2 spike glycoprotein mixed dataset. For each class the real-space cross-correlation was computed with 25 evenly spaced models from the closed state and the open state MD trajectories. (b) Distribution of particles in each class over the frames of the MD trajectory. (c) Precision per 3D class (left) and fraction of particles in each 3D class (right). (d) Initial models of each 3D class, only class 1 and 2 were selected for refinement. (e) Atomic models of the first and last conformation of the MD trajectory respectively. (f) Refined density maps for class 1 and 2. Visual inspection shows that class 1 resembles the target conformation, while class 2 resembles the initial conformation.

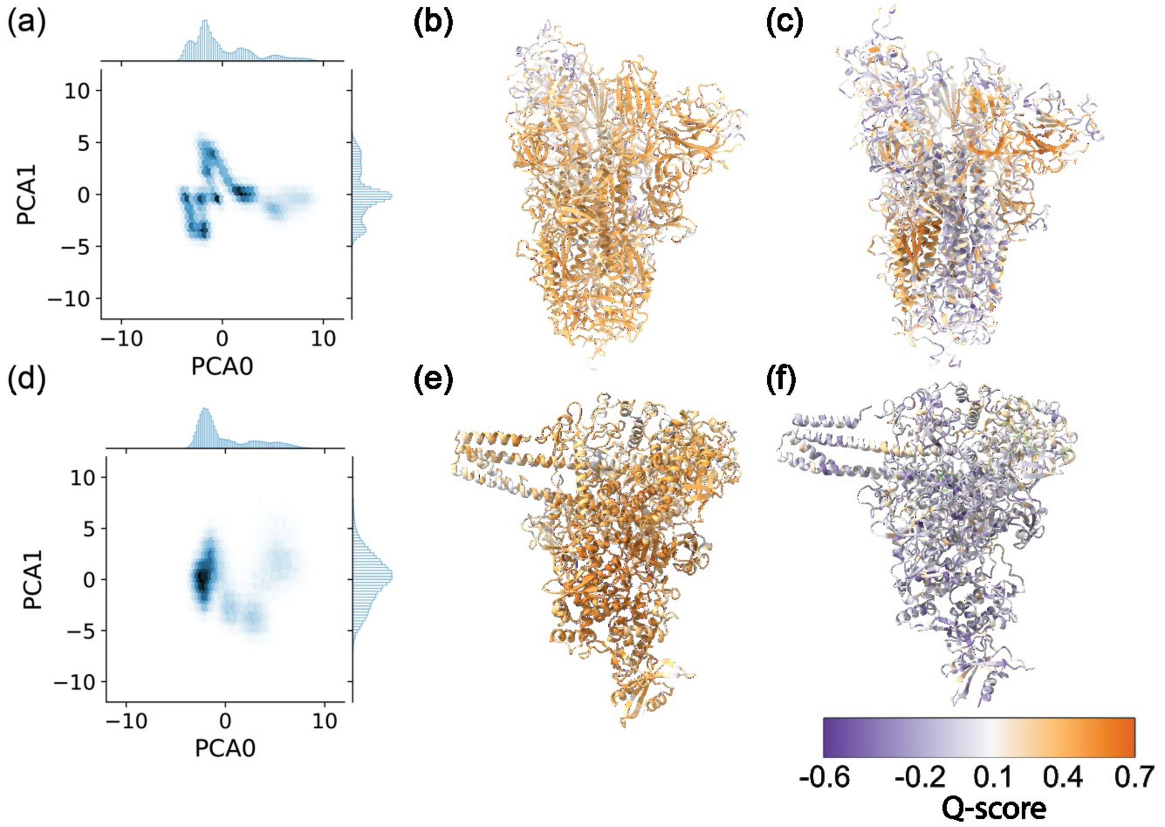

Figure S4. Further analysis of heterogeneous reconstruction of SARS-CoV-2 spike trimer and RTC. (a) Latent space of CryoDRGN training, visualised as a hexbin plot. This better shows the density in each region of the latent space. (b,c) Best and worst atomic model fit to sampled volume 46 of spike protein CryoDRGN training. (d) Hexbin visualisation of the latent space of the CryoDRGN training done for the RTC dataset. (e,f) Best and worst atomic model fit to sampled volume 43 for RTC complex CryoDRGN training.

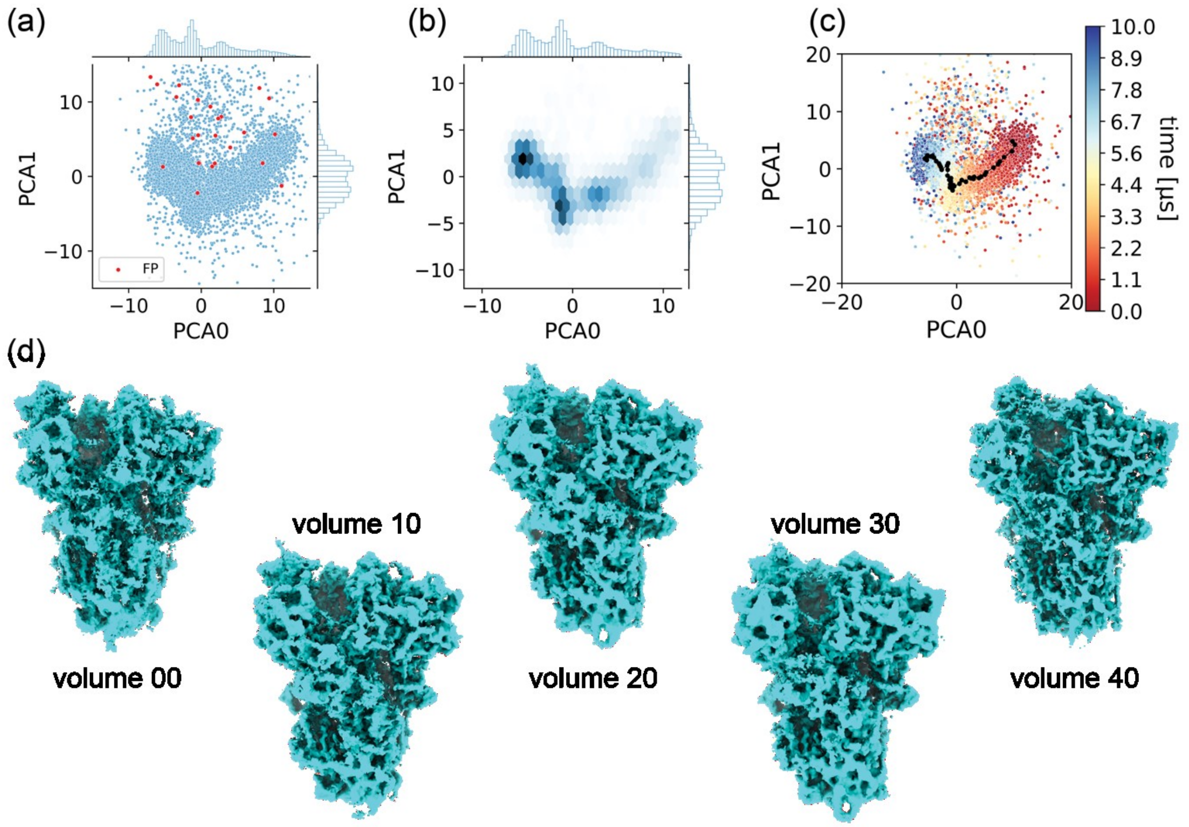

Figure S5. CryoDRGN training on the dose-fractionated dataset. (a) Scatterplot of the latent space, with false positive (FP) particles marked in red. (b) Hexbin plot of the latent space, showing it consists of a continuous region of density. (c) Latent space coloured by timepoint in the MD trajectory. (d) Example volumes generated from the averaged latent space coordinates in (c).

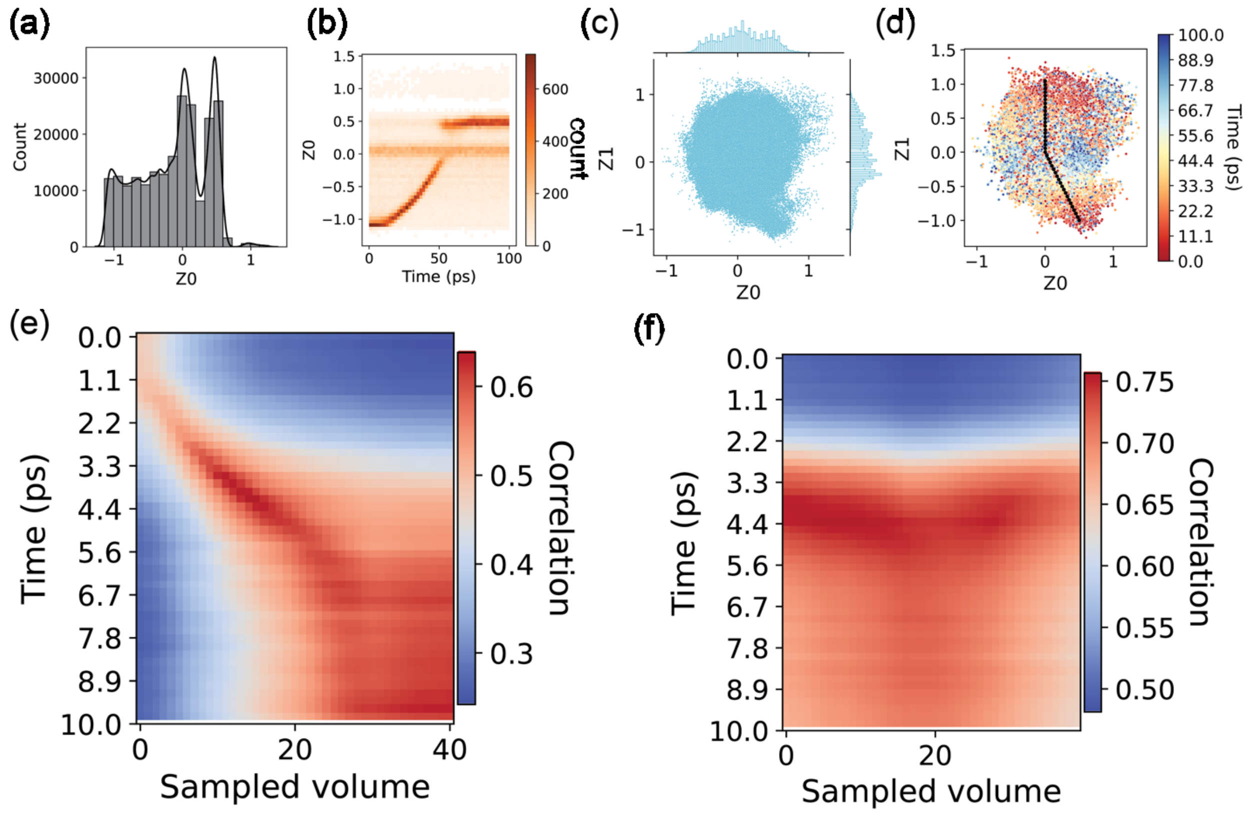

Figure S6. 3DFlex training for the SARS-CoV-2 spike monomer with zdim = 1 and zdim = 2. (a) Distribution of latent space coordinates (zdim = 1). (b) 2D histogram of the latent space coordinates and the timepoint in the MD trajectory of each particle. This plot shows a strong relation between the latent space coordinates and the trajectory. (c) Latent space from training with zdim = 2. (d) Latent space where each particle is coloured by its corresponding timepoint in the MD trajectory. Traversal through the latent space is plotted in black. (e) Real-space correlation heatmap between the MD trajectory and volumes sampled from the 1-dimensional latent space. (f) Real-space correlation heatmap for the 2-dimensional latent space.

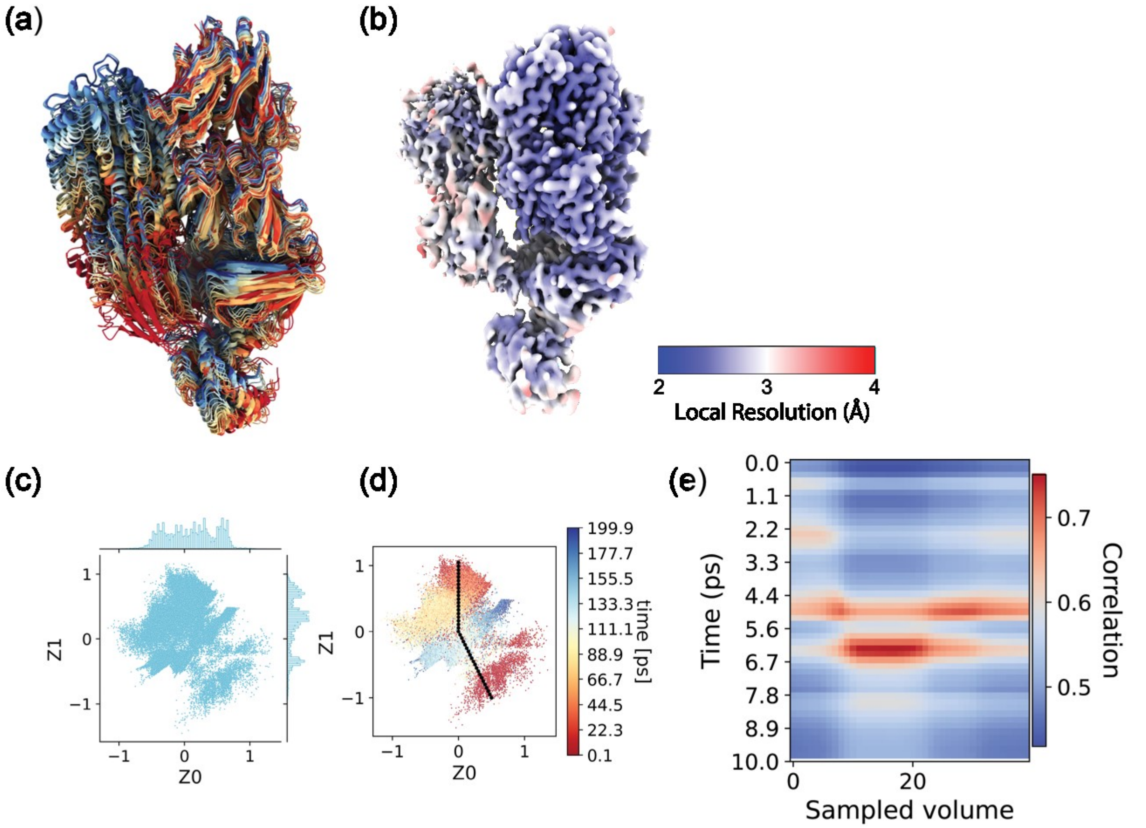

Figure S7. 3DFlex training for C3-C3b steered MD with zdim = 2. (a) Ensemble atomic model of the trajectory. Each model is coloured by the time in the MD simulation it originated from. (b) Reconstructed consensus density map, coloured by local resolution. (c) 2-dimensional latent space obtained. (d) Latent space coloured by timepoint each particle originated from in the MD trajectory. Traversal through the latent space used for sampling volumes is plotted in black. (e) Real-space map-to-model correlation heatmap between 50 states sampled from the MD trajectory and 41 sampled volumes.

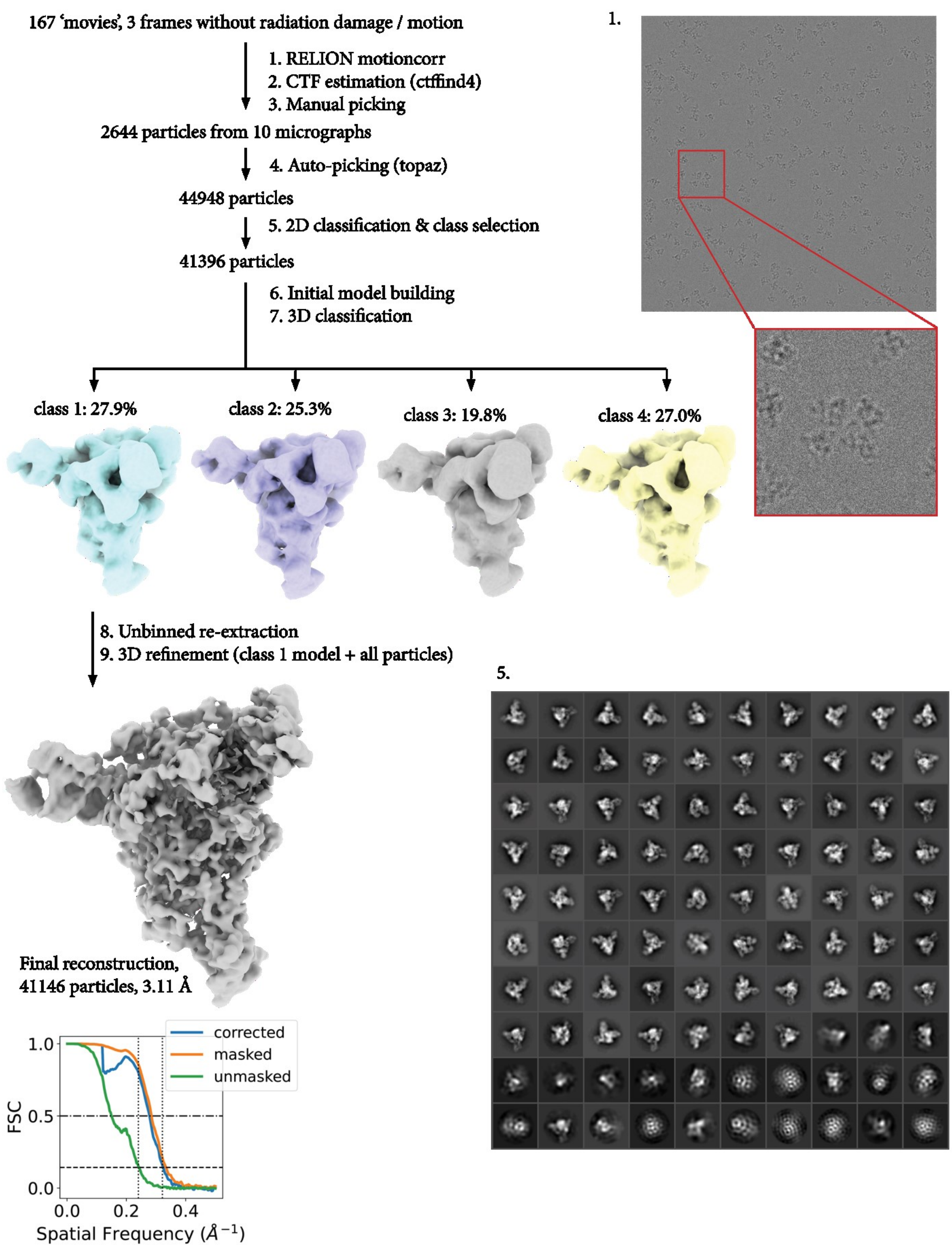

Figure S8. Processing workflow for SARS-CoV-2 RTC dataset based on DESRES-ANTON-13795965 MD trajectory.

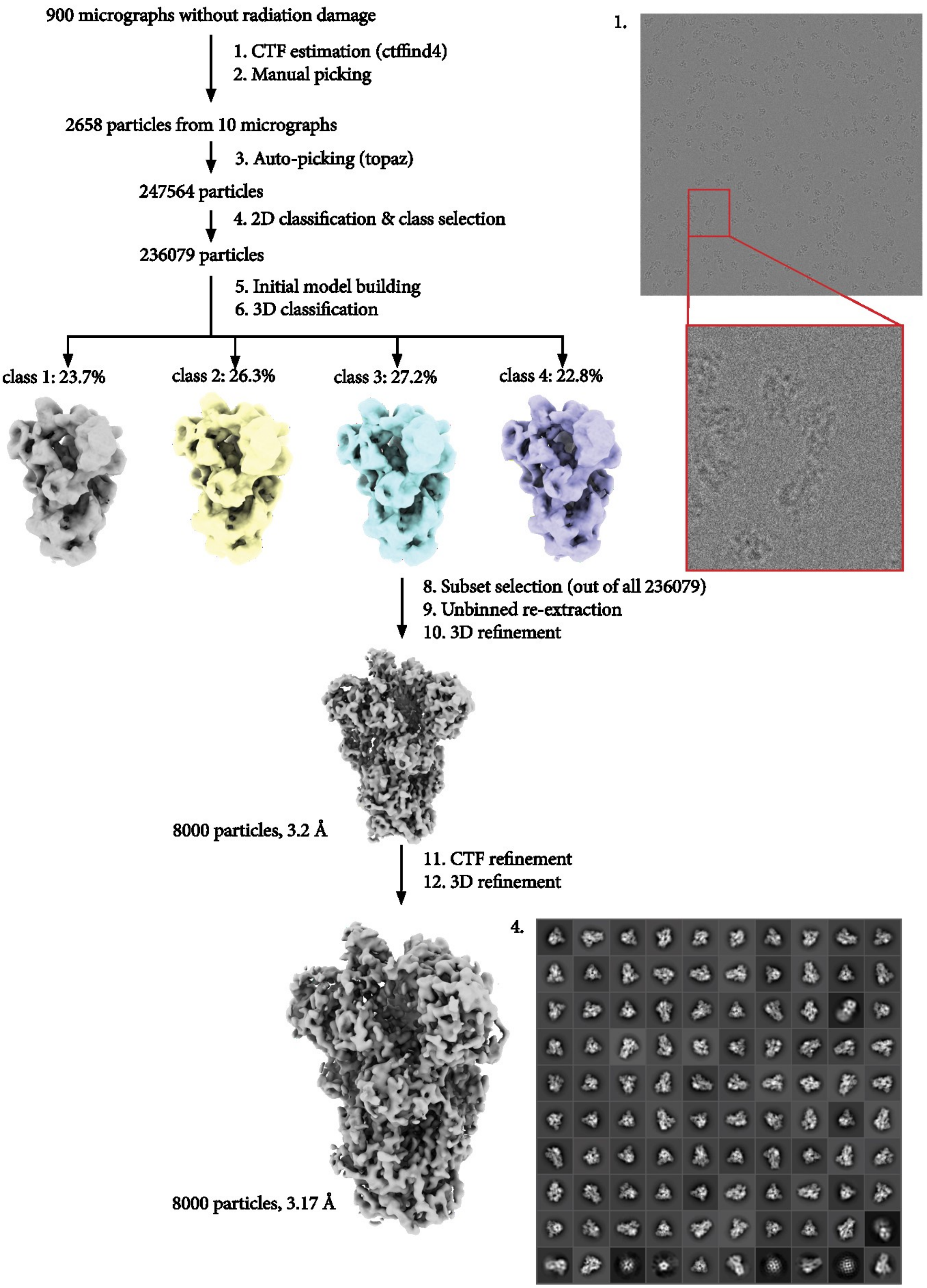

Figure S9. Processing workflow for SARS-CoV-2 spike glycoprotein dataset based on DESRES-ANTON-11021571 MD trajectory.

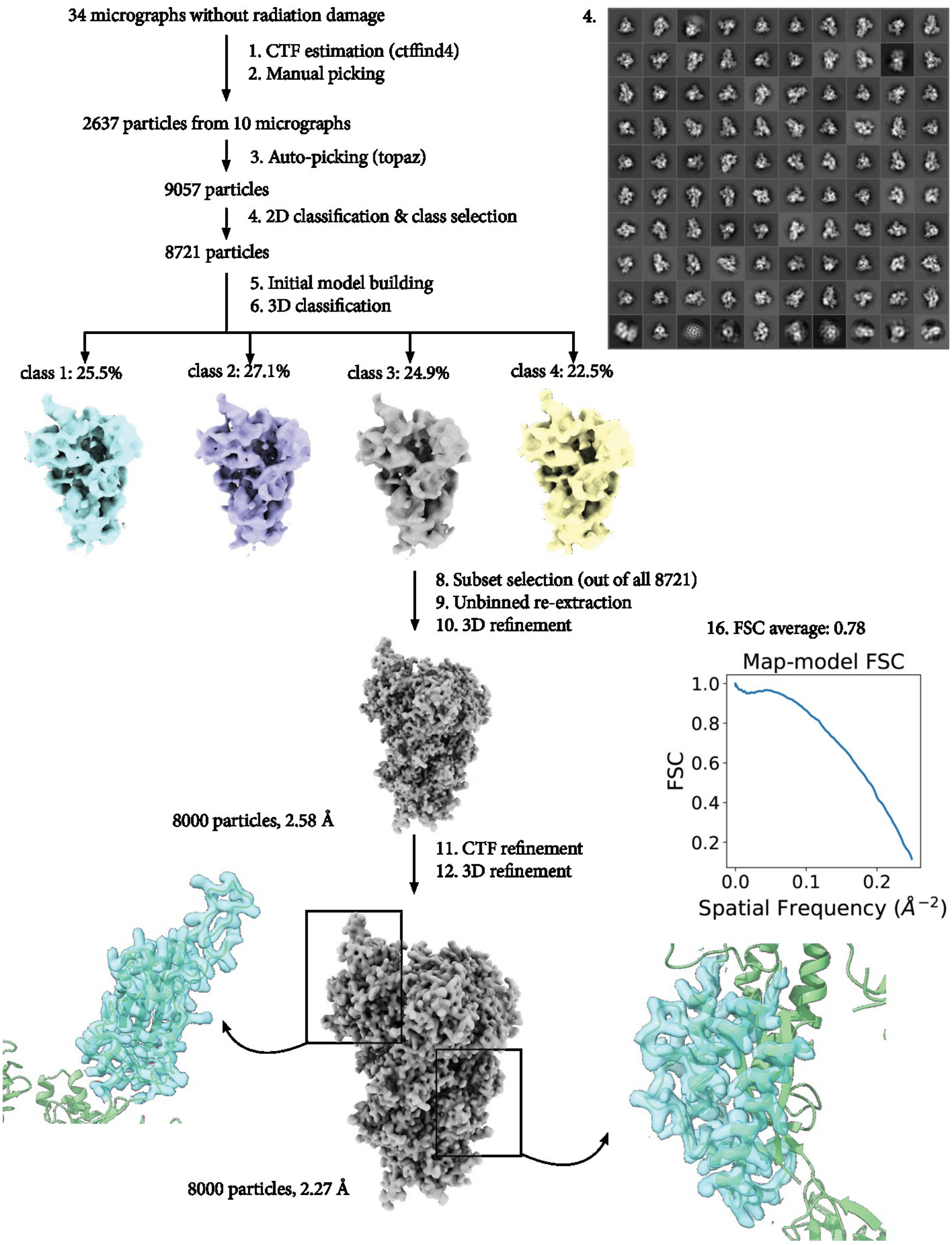

Figure S10. Processing workflow for SARS-CoV-2 spike glycoprotein dataset based on a single conformation from the DESRES-ANTON-11021571 MD trajectory.

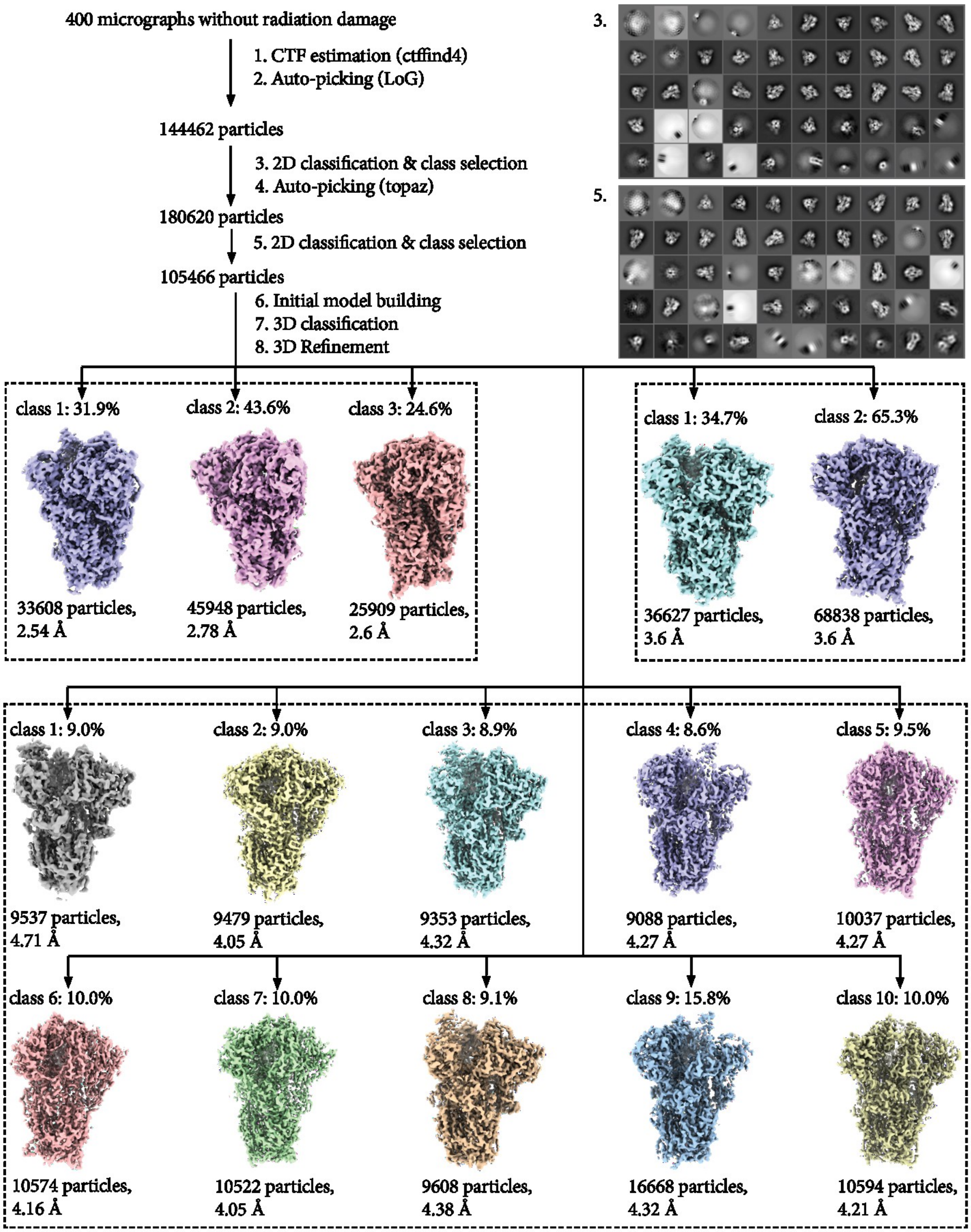

Figure S11. Processing workflow for mixed dataset based on the DESRES-ANTON-11021571 and DESRES-ANTON-11021566 trajectories. Density maps are shown for all classes after 3D classification with [2, 3, 10] classes and 3D refinement in RELION.

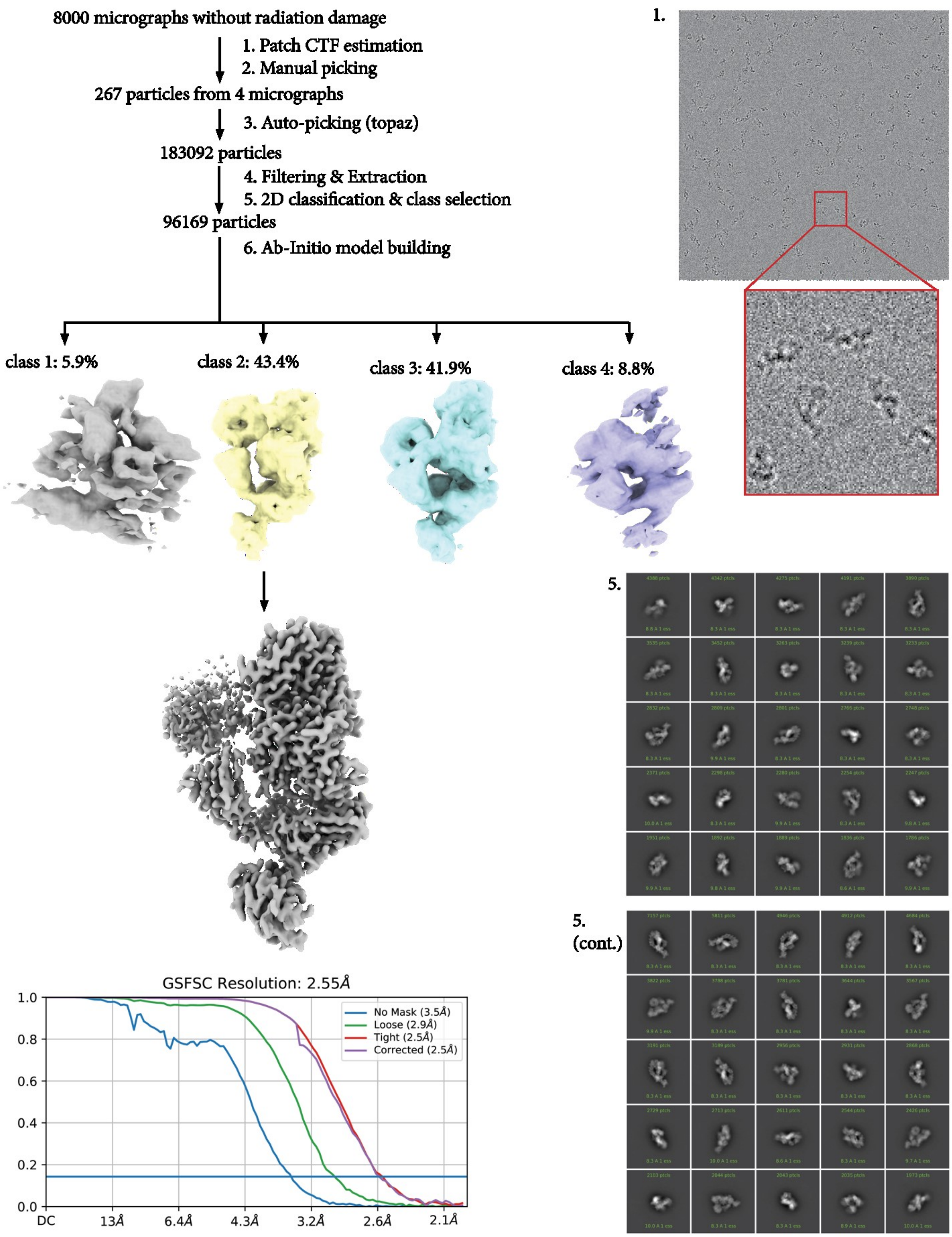

Figure S12. Processing workflow for C3 dataset based on steered MD simulation. Processing was done in CryoSPARC 4.2.1

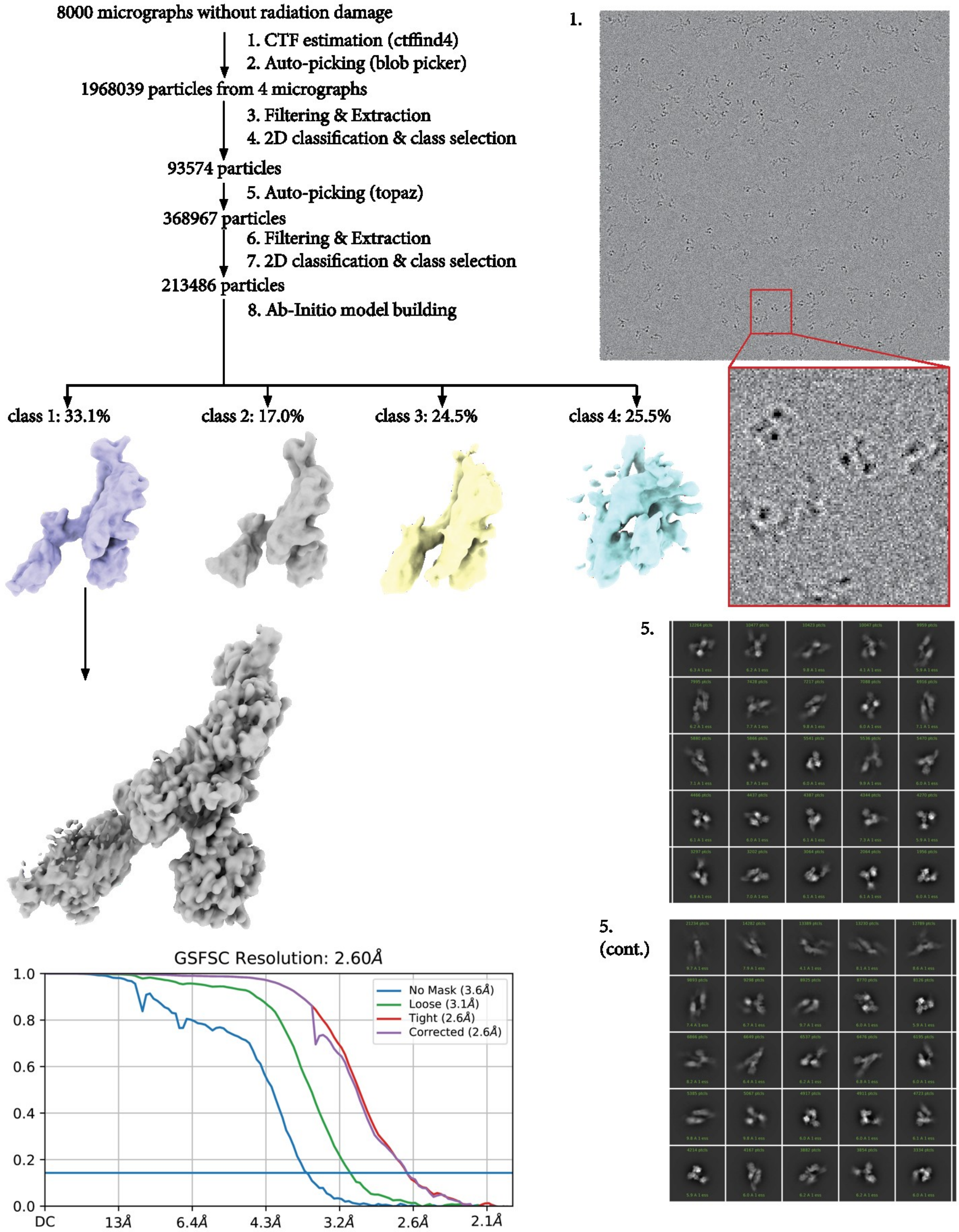

Figure S13. Processing workflow for single monomer SARS-CoV-2 spike glycoprotein based on steered MD simulation. Processing was done in CryoSPARC 4.2.1
